# Telomerase is required for glomerular renewal in kidneys of adult mice

**DOI:** 10.1038/s41536-022-00212-z

**Published:** 2022-02-11

**Authors:** Margo Montandon, Tynhinane Hamidouche, Lucile Yart, Lou C. Duret, Catherine Pons, Nicolas Soubeiran, Mélanie Pousse, Ludovic Cervera, Valérie Vial, Julien Fassy, Olivier Croce, Eric Gilson, Marina Shkreli

**Affiliations:** 1 Université Côte d’Azur (UCA), Centre National de la Recherche Scientifique (CNRS) UMR7284, Institut National de la Santé et de la Recherche Médicale (Inserm) U1081, Institute for Research on Cancer and Aging, Nice (IRCAN), Nice, 06107 France; 2 Université Côte d’Azur (UCA), Centre National de la Recherche Scientifique (CNRS) UMR7275, Institut de Pharmacologie Moléculaire et Cellulaire (IPMC), Valbonne, 06560 France; 3 International Laboratory in Hematology and Cancer, Shanghai Jiao Tong University School of Medicine/Ruijin Hospital/CNRS/INSERM/Nice University, Pôle Sino-Français de Recherche en Sciences du Vivant et Génomique, Shanghai Ruijin Hospital, Huangpu, Shanghai, 200025 P.R. China; 4 Department of Genetics, CHU Nice, Nice, 06202 France

**Keywords:** Regeneration, Adult stem cells, Podocytes, Toxin-induced nephropathy

## Abstract

Homeostatic renal filtration relies on the integrity of podocytes, which function in glomerular filtration. These highly specialized cells are damaged in 90% of chronic kidney disease, representing the leading cause of end-stage renal failure. Although modest podocyte renewal has been documented in adult mice, the mechanisms regulating this process remain largely unknown and controversial. Using a mouse model of Adriamycin-induced nephropathy, we find that the recovery of filtration function requires up-regulation of the endogenous telomerase component TERT. Previous work has shown that transient overexpression of catalytically inactive TERT (i-TERT^ci^ mouse model) has an unexpected role in triggering dramatic podocyte proliferation and renewal. We therefore used this model to conduct specific and stochastic lineage-tracing strategies in combination with high throughput sequencing methods. These experiments provide evidence that TERT drives the activation and clonal expansion of podocyte progenitor cells. Our findings demonstrate that the adult kidney bears intrinsic regenerative capabilities involving the protein component of telomerase, paving the way for innovative research toward the development of chronic kidney disease therapeutics.

## Introduction

Chronic kidney disease (CKD) is a global pandemic characterized by progressive renal function decline ultimately leading to end-stage renal disease, and represents an important risk factor for cardiovascular diseases and premature death^1^. The majority of kidney diseases that progress to CKD result from the depletion of glomerular visceral cells called podocytes. Historically, these highly specialized and quiescent epithelial cells have been thought to have a severely limited capacity for renewal^2,3^. However, several recent lines of evidence support the idea that podocytes can be at least partially renewed in the adult kidney following injury^4^.

Several lineage tracing studies have shed light on the source of renal progenitor cells, capable of supporting limited podocytes renewal in the postnatal kidney. These include the parietal epithelial cells (PECs) derived from the Bowman’s capsule^5,6^, and cells of renin lineage (CoRL) located in the juxtaglomerular apparatus^7–9^. Although these lineage tracing studies demonstrate the kidney’s modest capacity for intrinsic podocyte renewal, the filtration function of the kidney fails to fully recover in adult mice^10,11^. Additionally, the effectiveness of the documented potential progenitors to reconstitute podocytes as well as the molecular signals driving podocyte renewal remain unclear. One pathway that regulates podocytes in adults implicates non-canonical activity of telomerase^12^. Telomerase is a ribonucleoprotein complex that consists of TERT (the telomerase reverse transcriptase catalytic subunit) and TERC (the telomerase RNA subunit encoding the template sequence that is reverse transcribed by the telomerase enzyme to telomeres). Beside its well-established role in telomere synthesis, several studies demonstrated that TERT possesses non-canonical activities that foster cancer cell proliferation and adult stem/progenitor cells activation^13–17^. In the adult kidney, podocytes bear a high sensitivity to TERT non-canonical activity, an insight that comes from experimental models in which the catalytically inactive form of TERT (TERT^ci^)—which is unable to elongate telomeres—is conditionally overexpressed^12,18^. This study revealed that overexpression of TERT^ci^ in double transgenic mice (i-TERT^ci^ mice) induces reprogramming of podocyte into de-differentiated and proliferating cells. Intriguingly, after the transgenic TERT^ci^ is switch-off, there is a resurgence of quiescent and fully functional podocytes^12^.

To establish the role of TERT in kidney physiological regeneration, we used the Adriamycin-induced nephropathy model to cause a podocyte-specific insult and investigated the role of endogenous TERT in this context. We demonstrate that functional recovery of the kidney filtration barrier requires the upregulation of endogenous TERT expression, and identify signaling pathways involved in physiological regeneration. To assess the cascade of cellular events involved in podocyte renewal in the transgenic i-TERT^ci^ model, we carried out lineage tracing approaches using EdU-labeling strategies and an unbiased stochastic multicolor system for clonal analysis. We demonstrate that clonal expansion of progenitor cells gives rise to monoclonal glomeruli and repopulate the podocyte layer. In addition, we document the requirement of bi-allelic endogenous TERT to achieve successful recovery of the filtration function. Bulk RNA sequencing profiling further shed light on key molecular signals deployed in response to TERT, including the remodeling of the extracellular matrix, the Epithelial-to-Mesenchymal transition, and the activation of KRAS signaling. Altogether, our results reveal a central role for telomerase in modulating regenerative capabilities of the adult mammalian kidney.

## Results

### Renal filtration recovery in Adriamycin-induced nephropathy involves glomerular repair

**Fig. 1:**
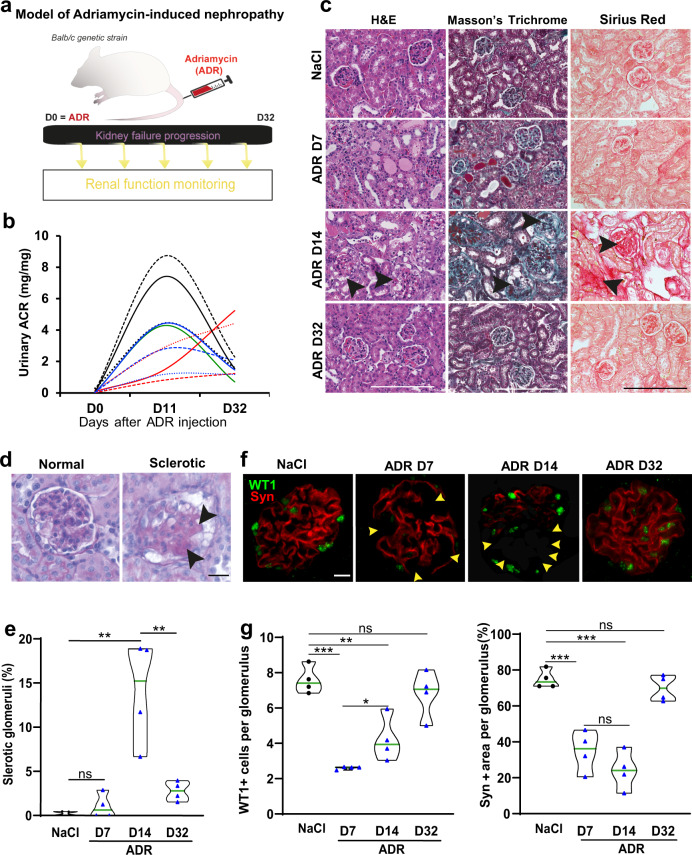
Renal filtration recovery in Adriamycin-induced nephropathy involves glomerular repair. **a** Schematic representation of the Adriamycin-induced nephropathy model. BALB/c mice were injected with a single dose of 12 mg/kg of Adriamycin (ADR) or saline (NaCl 0.9%) (day 0, D0), then euthanized 7 (D7), 14 (D14), or 32 days (D32) after injection. Proteinuria was monitored in the time course of the experiment for each individual mouse. **b** Kinetic analysis of kidney filtration function assessed by [albumin/creatinine ratio (ACR)] measurement in urine samples of ADR-injected female BALB/c mice (*n* = *10*), before injection (D0), 11 (D11), and 32 (D32) days after injection. Data are represented for each animal in (mg/mg). **c** H&E (left panels), Masson Trichrome (middle panels), and Sirius Red (right panels) stained kidney sections of BALB/c mice injected with saline (NaCl) or collected 7 (D7), 14 (D14), and 32 (D32) days after ADR injection. Scale bar = 200 µm. **d** Glomerular histology by PAS (Periodic Acid Schiff) from saline (Normal) or ADR-injected (Sclerotic) BALB/c mice. Scale bar = 30 µm. Arrowheads: sclerotic area. **e** Quantification of glomeruli with abnormal morphology (such as displayed in (**d**)) in kidney sections of BALB/c mice injected with saline (NaCl) or collected 7 (D7), 14 (D14), and 32 (D32) days after ADR injection (*n* = 4 for each group). For each animal, all glomeruli (about 150) on the whole kidney section were analyzed. Data are shown for each animal and the mean value for each group is shown as a green line. **p = 0.0035, and **p = 0.0098 by t-test for ADR-D14 versus saline and for ADR-D32 versus ADR-D14, respectively. **f** Double immunostaining for the podocyte-restricted markers Synaptopodin (Syn, red) and Wilms tumor protein 1 (WT1, green), in kidney section from saline or ADR-injected mice, 7 (D7), 14 (D14), and 32 days (D32) after ADR injection showing effacement of podocytes starting from 7 days post-ADR treatment (arrowheads) followed by marked normalization of podocytes at day 32. Scale bar = 15 µm. **g** Left panel: Quantification of the mean number of WT1 positive cells per glomerular cross section. ***p = 0.0001, and **p = 0.0037 by t-test for ADR-D7 and ADR-D14 versus saline respectively. *p = 0.0415 by *t*-test for ADR-D14 versus ADR-D7. Right panel: Quantification of the percentage of Synaptopodin positive area per glomerulus. ***p = 0.0006, and ***p = 0.0001 by *t*-test for ADR-D7 and ADR-D14 versus saline respectively. For each animal (*n* = 4 for each group), all glomeruli (about 150) on the whole kidney section were analyzed.

Adriamycin (ADR) nephropathy has been classically described as a rodent model of chronic kidney disease^19–21^. Nonetheless, spontaneous partial proteinuria remission following ADR injection in adult mice has been reported^6,22^. We hypothesized that renal filtration recovery following ADR-induced nephropathy can be explained by two distinct mechanisms; i) the filtration function is ensured by the remaining healthy podocytes that compensate for the impaired sclerotic glomeruli, ii) a sufficient proportion of damaged glomeruli are repaired upon a physiological process involving podocyte renewal. To test our hypothesis, we assessed the kinetic of disease progression following ADR-induced podocyte injury (Fig. 1a). We injected 10 male and 10 female BALB/c mice with a single dose of 12 mg/kg of ADR, and we subsequently monitored proteinuria as well as urinary albumin to creatinine ratio (ACR), a parameter that takes into account animal hydration. Both proteinuria and ACR monitoring revealed that ADR induced a kidney filtration dysfunction characterized by a severe protein leakage in the urine peaking at 11 days post-ADR injection (Fig. 1b, Supplementary Fig. 1). Filtration function improved over time and regressed almost completely 32 days after ADR injection in both male and female mice (Supplementary Fig. 1). Thus, only 5 to 25% of individuals retain a kidney filtration dysfunction 32 days after ADR injection characterized by either a proteinuria or an ACR level that exceed that observed 11 days after ADR injection (Fig. 1b, red curves, Supplementary Fig. 1). These data show that injection of a single dose of ADR in both male and female BALB/c mice induces massive leak of proteins into the urine that is followed by physiological recovery of filtration function in at least 70% of treated mice. To further examine the histological characteristics associated with kidney filtration function recovery in this model, we injected BALB/c female mice with a single dose of 12 mg/kg of ADR or with saline. The kidney histology was examined at 7, 14. and 32 days post-injection (Fig. 1c). Kidneys collected 14 days after ADR injection, corresponding to 3 days after the peak of proteinuria, displayed the characteristic abnormalities of human FSGS including cellular vacuolization, and interstitial fibrosis (Fig. 1c, arrows)^20^. Indeed, analysis of collagen deposits using Masson’s Trichrome and Sirius Red staining showed accumulation of extracellular matrix (ECM) components in the tubulointerstitial compartment and in glomeruli 14 days after ADR injection (Fig. 1c, arrows). While kidney filtration function started to improve at 14 days after injury, the prominent fibrosis observed at that time point remained associated with abnormally high levels of proteinuria (Supplementary Fig. 1). Examination of kidneys from mice sacrificed 32 days after ADR injection revealed that tubulointerstitial fibrosis and glomerular sclerosis strikingly regressed at that time point (Fig. 1c). In addition, further quantification of glomeruli with abnormal morphology associated with sclerosis and accumulation of ECM components showed a marked normalization at 32 days after ADR injection (Figs. 1d, e). Further examination of podocytes status revealed effacement of podocyte differentiation markers WT1 and synaptopodin starting from 7 days post-ADR treatment (Fig. 1f, g). Remission of proteinuria observed 32 days after ADR injection was associated with the normalization of podocytes (Fig. 1f, g). These processes were accompanied by a moderate but significant increase in cell proliferation, a hallmark of physiological regeneration in mammalian organs^23^, from day 7 after ADR injection that reached a peak 14 days after ADR injection (Supplementary Fig. 2). Noteworthy, cellular proliferation observed following ADR-induced injury does not appear to occur within the glomerular compartment (Supplementary Fig. 2). Collectively, these results suggest that kidney filtration function recovery is associated with glomerular repair and podocyte renewal. Therefore, we sought out to employ the ADR model to explore telomerase functions in the spontaneous regeneration of podocytes.

### Endogenous TERT is required for glomerular repair following ADR-induced nephropathy

**Fig. 2:**
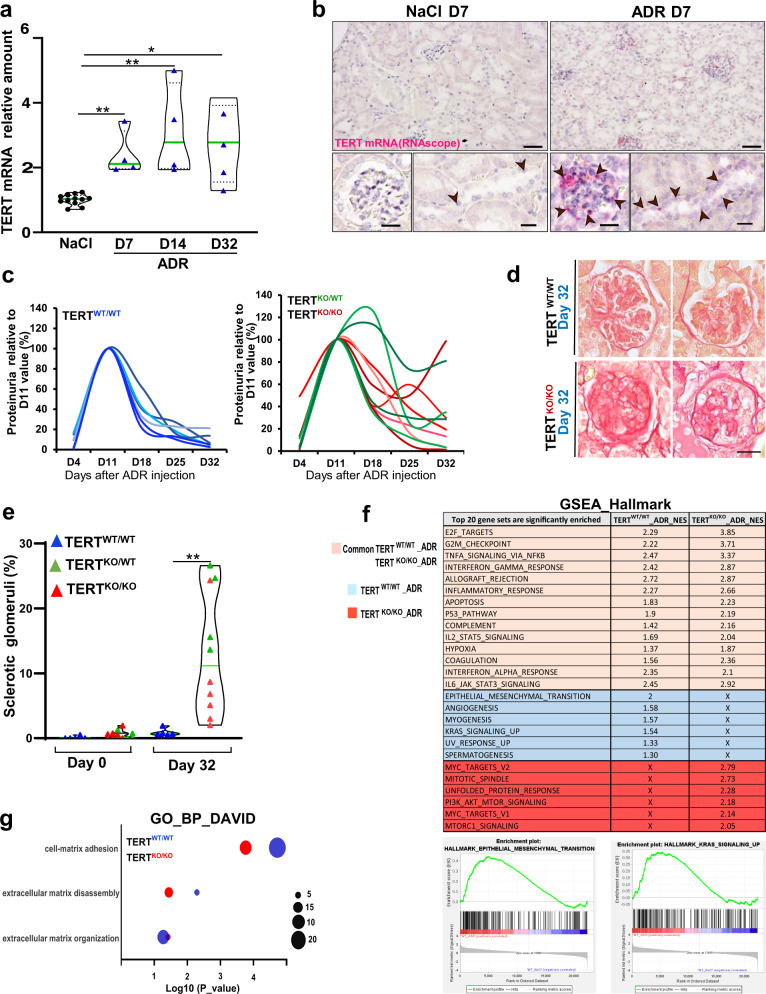
Endogenous TERT is required for glomerular repair following ADR-induced nephropathy. **a** *TERT* mRNA levels by RT-qPCR in whole kidneys from saline (NaCl, *n* = 12) and ADR-injected mice (ADR, *n* = 4 for each group) collected 7 (D7), 14 (D14), and 32 (D32) days after injection. Data are shown for each animal and the mean value for each group is shown as a green line. **p = 0.006 by t-test for ADR-D7 versus saline. **p = 0.005 by t-test for ADR-D14 versus saline. *p = 0.014 by t-test for ADR-D32 versus saline. **b** In situ hybridization for *TERT* mRNA using RNAscope on kidney sections from saline and ADR-injected mice sacrificed 7 days after injection. Upper panels, scale bar = 50 µm. Lower panels, scale bar = 20 µm. Arrowheads: cells expressing detectable amounts of TERT mRNA. **c** Kinetic analysis by Bradford assay of proteinuria after ADR injection of female BALB/c N10 control mice (TERT^WT/WT^, blue) (left panel) and female BALB/c N10 mice carrying a full invalidation (TERT^KO/KO^, red) or heterozygous invalidation (TERT^KO/WT^, green) (right panel) of *TERT*. **d** Glomerular histology by Sirius Red from ADR-injected TERT^WT/WT^ and TERT^KO/KO^ mice 32 days after ADR injection. Scale bar = 20 µm. **e** Quantification of glomeruli with abnormal morphology (such as displayed for TERT^KO/KO^ mice in (**d**)), in kidney sections from TERT^WT/WT^ (*n* = 6, blue triangles), TERT^KO/WT^ (*n* = 4, green triangles) and TERT^KO/KO^ (*n* = 6, red triangles) mice collected before ADR injection (Day 0), and 32 days after ADR injection (Day 32). For each animal, all glomeruli (about 150) on the whole kidney section were analyzed. Data are shown for each animal and mean value for each group is shown as a green line. **p = 0.0071 by *t*-test for *TERT* knockout versus control mice at day 32. **f** Comparison of the top 20 enriched gene signatures found by Gene Set Enrichment Analysis (GSEA) Hallmark in TERT^WT/WT^ and TERT^KO/KO^ mice 18 days after ADR injection. Common gene signatures between TERT^WT/WT^ and TERT^KO/KO^ mice are highlighted in tan, gene signatures only enriched in TERT^WT/WT^ mice are highlighted in blue, and gene signatures only enriched in TERT^KO/KO^ mice are highlighted in red. NES stands for normalized enrichment score. Enrichment profiles of Epithelial-to-Mesenchymal transition and KRAS signaling UP are shown. **g** Database for Annotation, Visualization and Integrated Discovery (DAVID) analysis showing the status of GO-term (Biological Process) related to the extracellular matrix (ECM) in TERT^WT/WT^ (blue) and TERT^KO/KO^ (red) mice 18 days after ADR injury. The size of the circles is proportional to the number of genes attributed to each GO-term.

To determine whether the repair process triggered by ADR-induced podocyte injury was associated with endogenous reactivation of TERT expression, we performed RT-qPCR on whole kidneys. This analysis revealed significant upregulation of *TERT* mRNA expression 7 days after ADR injection that was maintained in the time course of the experiment (Fig. 2a). Interestingly, the mice with persistent kidney filtration dysfunction at 32 days after ADR injection, as assessed by proteinuria and ACR analysis, displayed significantly lower levels of *TERT* mRNA when compared to the mice that recovered properly (Supplementary Fig. 3). Further in situ analysis using the highly sensitive RNA detection technique RNAscope revealed that TERT upregulation was confined to glomeruli and clusters of cells located within tubular segments of the nephron at 7 days post-ADR treatment (Fig. 2b). Noteworthy, podocytes did not appear to be the source for increased *TERT* mRNA level in glomeruli (Supplementary Fig. 4). These results reveal a specific upregulation of TERT in nephron compartments at an early time point following ADR-induced nephropathy.

To test the requirement of endogenous TERT in the kidney recovery response, we used TERT constitutive knockout (KO) mice in the ADR model of physiological kidney repair. Telomeres of laboratory mouse strains are longer than their human counterparts, and although telomere synthesis is abrogated in TERT KO mice, phenotypes associated with telomere attrition are not detectable in first generations TERT KO mice^24,25^. Thus, both heterozygous (TERT^KO/WT^) and first-generation (G1) homozygote TERT KO (TERT^KO/KO^) mice do not harbor any defect in proliferating tissues and are fertile. To test the ability of TERT KO mice to recover following ADR-induced kidney injury, we injected females G1 TERT^KO/KO^ and TERT^KO/WT^ mice, and females TERT^WT/WT^ littermates, all obtained from interbreeding of TERT^KO/WT^ BALB/c N10 mice, with a single dose of 12 mg/kg of ADR, as performed on BALB/c mice. Monitoring of proteinuria following ADR injection revealed a similar peak of proteinuria 11 days after ADR injection in TERT^KO/KO^, in TERT^KO/WT^, and in their TERT^WT/WT^ littermates (Fig. 2c). However, in contrast to control TERT^WT/WT^ mice, both TERT^KO/KO^ and TERT^KO/WT^ mice showed a more heterogeneous recovery of kidney filtration function, with latent proteinuria levels significantly higher 18 days post-ADR treatment (Fig. 2c, Supplementary Fig. 5).

To determine if the perturbed kinetic of recovery observed in TERT^KO/KO^ and TERT^KO/WT^ mice was associated with changes in the balance between regeneration and scarring following injury, we examined ECM deposition 32 days after ADR injection (Fig. 2d). Such analysis showed that TERT^KO/KO^ and TERT^KO/WT^ mice display an increased prevalence of sclerotic glomeruli when compared to TERT^WT/WT^ mice (Fig. 2e). Those results show that invalidation of endogenous *TERT* results in an increased number of sclerotic glomeruli following podocyte injury, suggesting a role for endogenous TERT in kidney homeostatic repair. Importantly, the increased incidence of glomerular scarring in TERT^KO/KO^ mice was not associated with the shortening of mean telomere DNA length (Supplementary Fig. 6). Overall, these results suggest that endogenous TERT expression is required for kidney filtration recovery and ECM remodeling, independently of its catalytic activity in telomere elongation, to achieve glomerular repair following ADR-induced nephropathy.

To gain insight into the molecular processes targeted by TERT non-canonical functions upon glomerular repair, we performed high-throughput sequencing (bulk RNA-seq) on kidneys collected from TERT^KO/KO^ mice upon the recovery period, i.e. 18 days after ADR-injection. Comparison of TERT^KO/KO^ and TERT^WT/WT^ transcriptomes in saline-treated mice showed minimal changes, but suggested that TERT deficiency perturbed basal activation of several pathways including mTORC1, Wnt, and Notch signaling (Supplementary Figure 7). Further GSEA analysis of ADR-treated mice interestingly revealed that TERT deficiency precludes proper activation of signaling pathways, including Epithelial-to-Mesenchymal transition (EMT) and KRAS signaling, upon glomerular repair (Fig. 2f). In addition, assessment of Gene Ontology term (GO term) using Database for Annotation, Visualization and Integrated Discovery (DAVID) analysis highlighted impairment of extracellular matrix (ECM) remodeling upon deletion of *TERT* (Fig. 2g). Examination of the status of metalloproteinases (MMPs) revealed that *TERT* deletion prevented the upregulation of MMP-24, MMP-12, MMP-10, and MMP-3 that is normally observed in TERT^WT/WT^ mice upon glomerular repair following ADR injury (Supplementary Fig. 8). Altogether, these results highlight key signaling pathways involved in a TERT-dependent regenerative program following ADR-induced injury.

### Catalytically-inactive TERT overexpression triggers podocyte renewal from a non-podocyte source

**Fig. 3:**
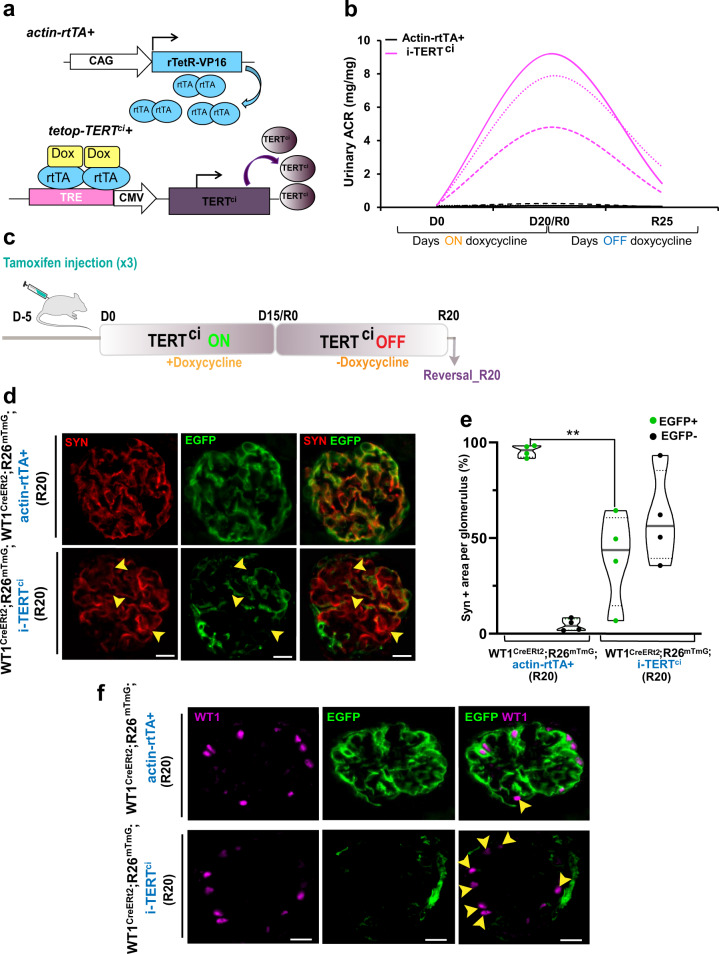
Catalytically-inactive TERT overexpression triggers podocyte renewal from a non-podocyte source. **a** Schematic of the inducible-TERT^ci^ (i-TERT^ci^) bi-transgenic system. The actin-rtTA+ transgene encodes the reverse tetracycline repressor (rTetR) fused to the transcription activation C-terminal domain of virion protein 16 (VP16) of herpes simplex virus (HSV). The resulting hybrid transactivator, called reverse tetracycline-controlled transactivator (rtTA), stimulates promoters fused to tetracycline operator (tetO) sequences in the presence of the inducer doxycycline (Dox). A CAG (cytomegalovirus (CMV) early enhancer/chicken beta-actin) promoter drives widespread expression of rtTA in the mouse. On the second tetop-TERT^ci^ + transgene, the sequence encoding TERT^ci^ is under the control of a tetracycline-responsive promoter element (TRE), composed of seven tetO sequences, that is linked to the CMV early enhancer element. In the presence of the tetracycline analog doxycycline, provided into the drinking water of the animals, high expression of TERT^ci^ is induced in i-TERT^ci^ mice. **b** Kinetic analysis of kidney filtration function assessed by [albumin/creatinine ratio (ACR)] measurement from single transgenic actin-rtTA + (*n* = 3, black) and i-TERT^ci^ (*n* = 3, pink) mice. Data are shown for each animal. **c** Schematic representation of the experimental design. Tamoxifen was injected 5 days prior to doxycycline treatment to induce permanent EGFP tagging of mature podocytes. Transient TERT^ci^ overexpression (TERT^ci^ ON) in i-TERT^ci^ mice was then induced for 15 days (D15) by the mean of doxycycline treatment, then the mice were subsequently submitted to a reversal period (TERT^ci^ OFF) for 20 days (R20) during which proteinuria gradually regressed. **d** Double immunostaining for Synaptopodin (SYN, red) and EGFP (green) in kidney sections from WT1^CreERt2^;R26^mTmG^;actin-rtTA+ control (Upper panels) and WT1^CreERt2^;R26^mTmG^;i-TERT^ci^ experimental mice (lower panels) euthanized 20 days after the reversal period. Scale bars = 20 µm. Arrow heads show differentiated podocytes (SYN + ) devoid of EGFP signal. **e** Quantification of data in (d). Percentage of SYN + area that do (EGFP + ) and do not (EGFP-) overlap with EGFP signal in glomeruli of WT1^CreERt2^;R26^mTmG^;actin-rtTA+ control mice (*n* = 4) and WT1^CreERt2^;R26^mTmG^;i-TERT^ci^ experimental mice (*n* = 4) sacrificed 20 days after the stop of doxycycline treatment (R20). Data are shown for each animal and the mean value for each group is shown as a grey line. For each animal, all glomeruli (about 150) on the whole kidney section were analyzed. **p = 0.0019 by t-test for WT1^CreERt2^;R26^mTmG^;i-TERT^ci^ vs. WT1^CreERt2^;R26^mTmG^;actin-rtTA+ mice. **f** Double immunostaining for EGFP (green) and the nuclear podocyte-restricted marker Wilms’ Tumor Protein 1 (WT1, magenta), in kidney sections from WT1^CreERt2^;R26^mTmG^;actin-rtTA+ control (Upper panels), and WT1^CreERt2^;R26^mTmG^;i-TERT^ci^ experimental mice (Lower panels) sacrificed 20 days after the stop of doxycycline treatment. Scale bar = 20 µm. Arrowheads show differentiated podocytes (WT1 + ) devoid of EGFP signal.

We next aimed to better dissect the cellular mechanisms that contribute to TERT-dependent podocyte renewal using a mouse model of catalytically inactive TERT (TERT^ci^) (Fig. 3a). We previously showed in i-TERT^ci^ mice that conditional and ubiquitous overexpression of TERT^ci^ impaired in its telomere elongation function, causes a dramatic effect on kidney podocytes proliferation that ultimately leads to a collapsing glomerulopathy reminiscent of human collapsing FSGS features^12^. The subsequent podocyte effacement results in severe proteinuria, a robust phenotype observed specifically in i-TERT^ci^ mice independently of their background (Supplementary Fig. 9)^12^. Importantly, this phenomenon is reversible following the switch-off of transgenic TERT^ci^ expression, and this reversibility is characterized by a marked improvement of kidney filtration function and re-expression of podocytes differentiation markers (Fig. 3b)^12^. However, the cellular mechanisms deployed to repair the glomerular compartment following a pulse of TERT^ci^ expression remain cryptic.

We first sought to determine whether renewed podocytes were emerging from simple podocyte duplication or from an external source of progenitor cell using a podocyte lineage tracing model (Fig. 3c, Supplementary Fig. 10). We first assessed the efficiency of EGFP-podocyte labeling following tamoxifen injection of WT1^CreERt2^;R26^mTmG^ control mice. Toward that goal, we performed double-immunostaining for EGFP and Synaptopodin that correspond to differentiated podocytes, in whole kidney paraffin sections and established the overlap between both signals. This analysis revealed the EGFP labeling of 95.7% (± 0.94%) of the Synaptopodin positive (Syn + ) area, defined as a threshold of EGFP expression for the downstream experiments (Supplementary Fig. 10).

We next performed podocyte lineage tracing following TERT^ci^ pulse and reversal, in WT1^CreERt2^;R26^mTmG^;i-TERT^ci^ compound mice (Fig. 3c, Supplementary Fig. 10). In this experiment, the EGFP permanent labeling of mature podocytes was induced with tamoxifen treatment prior TERT^ci^ induction (TERT^ci^ ON) (Fig. 3c, Supplementary Fig. 10). Therefore, we assessed the prevalence of EGFP + area within Syn+ area at the end of the reversal period. This analysis revealed a high proportion of differentiated podocytes (Syn+ positive cells) devoid of EGFP + signal in kidneys of WT1^CreERt2^;R26^mTmG^;i-TERT^ci^ mice reversed (Fig. 3d, arrows). Thus, while EGFP tagging of podocytes remained stable over the time of the experiment in control mice with 95.5% (± 0.82%) of Syn+ area overlapping with EGFP signal, 59.7% (± 14.3%) of differentiated podocytes observed after TERT^ci^-enforced renewal were devoid of EGFP signal (Fig. 3e). Those results show that about half of differentiated podocytes observed after a TERT^ci^ pulse in the adult kidney, ensue from cells that do not derive from initially EGFP-labeled podocytes. Double staining of kidney sections for EGFP and the transcription factor Wilms tumor protein 1 (WT1), expressed in mature podocytes, confirmed this observation (Fig. 3f, arrows). These data indicate that renewed podocyte do not emerge from podocyte duplication, suggesting that they come from an external source of progenitor cells.

### Tubular epithelial cells proliferate following a TERT^ci^ pulse

**Fig. 4:**
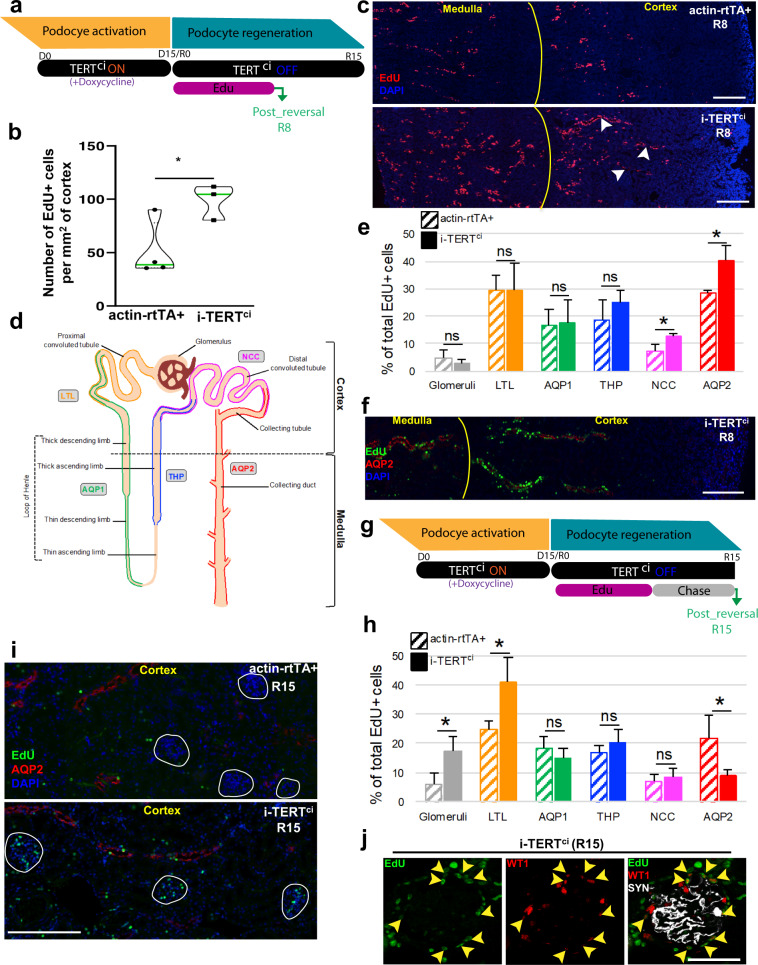
Tubular epithelial cells proliferate following a TERT^ci^ pulse. **a** Schematic representation of TERT^ci^-induced podocyte renewal experiment with EdU treatment upon the early phase of reversal (R0-R8). i-TERT^ci^ and actin-rtTA+ control mice were treated for 15 days (D15) with doxycycline to induce transient TERT^ci^ overexpression (TERT^ci^ ON) in i-TERT^ci^ mice. The thymidine analogue EdU was administrated in the drinking water of the animals from R0 to R8, and kidneys were collected at R8. **b** Number of EdU positive cells per millimeter square of cortical area in actin-rtTA+ control (*n* = 4) and i-TERT^ci^ mice (*n* = 3), in kidneys collected 8 days after stopping doxycycline treatment. Data are shown for each animal and mean value for each group is shown as a green line. *p = 0.020 by t-test for i-TERT^ci^ vs. actin-rtTA+ control mice. **c** Immunostaining for the thymidine analogue EdU (red) in kidney sections from actin-rtTA+ control (upper panel), and i-TERT^ci^ (lower panel) mice showing the emergence of arrays of EdU+ cells within the kidney inner cortex of i-TERT^ci^ mice (arrowheads). Scale bar = 300 µm. **d** Schematic representation of the adult mammalian nephron showing markers of its different sections that were used to locate EdU+ cells. **e** Quantification of EdU+ cells distribution within the different sections of the nephron in kidneys collected at the end of the EdU treatment (R8). Percentage of EdU+ cells located within the glomeruli or within the sections of the nephron expressing the Lotus Tetragonolobus Lectin (LTL), the water channel aquaporin 1 (AQP1), the Tamm-Horsfall glycoprotein (THP), the sodium-chloride symporter (NCC) or the water channel aquaporin 2 (AQP2) in actin-rtTA+ control (*n* = 4) and i-TERT^ci^ (*n* = 3) mice. Data are represented as mean ± SEM. *p = 0.015, and *p = 0.026 by t-test for i-TERT^ci^ versus control mice in NCC and AQP2 sections respectively. **f** Double immunostaining for EdU (green), and the water channel aquaporin 2 (AQP2, red), in a kidney section from an i-TERT^ci^ mouse euthanized 8 days after switching-off transgenic TERT^ci^ expression and treated with EdU drinking water for 8 days preceding euthanasia. Scale bar = 250 µm. **g** Schematic representation of EdU chase experiment upon TERT^ci^-induced regeneration. i-TERT^ci^ and actin-rtTA+ control mice were treated for 15 days (D15) with doxycycline to induce transient TERT^ci^ overexpression (TERT^ci^ ON) in i-TERT^ci^ mice. EdU was administrated in mice drinking water upon the first 8 days of reversal, and the mice were then switched to normal drinking water for 7 days until kidney collection (R15). **h** Quantification of EdU+ cells distribution within the different sections of the nephron after 7 days of EdU chase. Percentage of EdU+ cells located within the glomeruli or within LTL, AQP1, THP, NCC or AQP2 sections of the nephron in actin-rtTA+ control (*n* = 4) and i-TERT^ci^ (*n* = 3) mice. Data are represented as mean ± SEM. *p = 0.034, *p = 0.041, and *p = 0.050 by t-test for i-TERT^ci^ vs. actin-rtTA+ control mice in glomeruli, and in LTL and AQP2 sections respectively. **i** Double immunostaining for EdU (green), and the water channel aquaporin 2 (AQP2, red), in kidney sections from actin-rtTA+ control and i-TERT^ci^ mice sacrificed 15 days after switching-off transgenic TERT^ci^ expression and treated with EdU drinking water during the early phase of reversal (R0-R8). Glomeruli are lined by white circles. Scale bar = 200 µm. **j** Triple immunostaining for EdU (green), the nuclear podocyte-restricted marker Wilms’ Tumor Protein 1 (WT1, red) and the podocyte-restricted actin-associated protein synaptopodin (SYN, white) in a kidney section from an i-TERT^ci^ mouse euthanized after 7 days of EdU chase (R15) showing differentiated podocytes that incorporated EdU (arrowheads).

A common feature of regeneration in adult tissue or organ is the activation of quiescent adult stem cells, or differentiated cells, to govern the generation of progenitor cells that rapidly divide and give rise to terminally differentiated cells^26^. Hence, we sought to use the i-TERT^ci^ mouse model to chase the origin of podocyte progenitors. Toward that goal, we first characterized the kinetic of cell proliferation upon TERT^ci^-induced podocyte renewal. Dividing cells were labeled using the administration of the thymidine analogue EdU in the drinking water of mice along the time course of the reversal period (Fig. 4a). Kidneys were then collected upon the early reversal period, i.e. 8 days after switching-off TERT^ci^ expression (R8), a time point that corresponds to the early phase of proteinuria remission (Supplementary Fig. 11). Examination of EdU distribution within whole kidney sections of control mice revealed a basal level of cell proliferation within the kidney tubules (Fig. 4b, c). This result suggests that tubular epithelia undergo homeostatic turnover in steady-state conditions. Assessment of EdU distribution in the whole kidney section following a TERT^ci^ pulse revealed the specific appearance of arrays of proliferative cells within cortical tubular segments in i-TERT^ci^ mice (Fig. 4c, arrows). While those arrays of EdU+ cells represented a limited fraction of all EdU+ cells observed in whole kidney sections of i-TERT^ci^ mice (Fig. 4b, Supplementary Fig. 11), they were never observed in cortical tubular segments of control mice. To further determine the location of EdU+ cells within the nephron, we performed co-immunostaining for EdU and markers of the different sections of the nephron (Fig. 4d). Such analysis revealed that the arrays of EdU+ cells specifically observed in kidneys of i-TERT^ci^ mice were mainly located within AQP2 + collecting tubules and spread toward the NCC + distal convoluted tubules (Figs. 4e, f). Noteworthy, the number of EdU+ cells in glomeruli of i-TERT^ci^ mice remained low and similar to that observed in control actin-rtTA+ animals (Fig. 4e). Those results show that induction of cell proliferation right after silencing TERT^ci^ overexpression is mainly observed in the distal convoluted and collecting tubules, but remains low in glomeruli.

To further characterize the fate of EdU-labeled cells after the early phase of reversal, we performed a pulse-chase experiment. EdU was administrated in mice drinking water upon the first 8 days of reversal, and the mice were then switched to normal drinking water for 7 days until kidney collection (R15) (Fig. 4g). Analysis of EdU+ cells in this setting revealed an important depletion of those cells within the AQP2-expressing section of the nephron in i-TERT^ci^ mice at R15 when compared to R8 (Fig. 4h, Supplementary Fig. 11). This 77% drop in the number of EdU+ cells within the AQP2 + segment suggests that proliferation of tubular AQP2 + cells observed after TERT^ci^ switch-off is followed by an extensive renewal of this portion of the nephron. Nonetheless, specific loss of these arrays of EdU+ cells in AQP2 + segment did not involve apoptosis as assessed by TUNEL assay (Supplementary Fig. 12).

We next analyzed the distribution of EdU+ cells after the chase period and we observed a specific and significant enrichment of proliferative cells in glomeruli and proximal convoluted tubules of i-TERT^ci^ mice (Fig. 4h, i, Supplementary Fig. 11). Part of those glomerular EdU+ cells, specifically observed in glomeruli of i-TERT^ci^ mice after the chase period, expressed the podocyte differentiation marker WT1 (Fig. 4j). These tracing methods indicate that cell proliferation is initiated in the inner cortex at the early stages of the TERT^ci^ reversal period, corresponding to the distal convoluted and collecting tubules of the nephron and is further observed in the glomeruli and proximal tubules located within the outer cortex after the chase period. These results demonstrate the dynamic epithelial cell proliferation within the different segments of the nephron throughout the TERT^ci^ reversal period, suggesting the activation of transit-amplifying cells in response to TERT^ci^ expression.

### Monoclonal glomeruli emerge following a telomerase pulse in the adult kidney

**Fig. 5:**
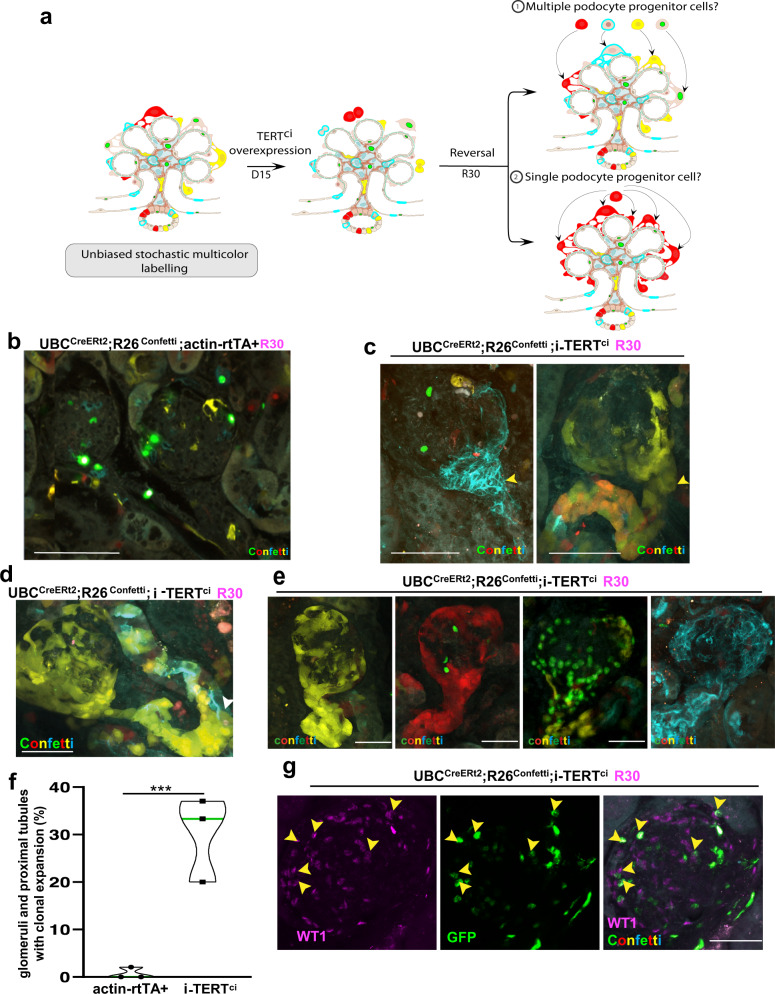
Clonal expansion of mono-colored cells invade the glomerulus compartment following a TERT^ci^ pulse in the adult kidney. In this figure, spectral confocal microscopy images depicting the overlay of the 4 Fluorescent Proteins (FP) are indicated by “Confetti”. **a** Schematic of unbiased approach used to track progenitor cells in i-TERT^ci^ mice. Adult UBC^CreERt2^;R26^Confetti^;i-TERT^ci^ mice were treated with sub-optimal doses of tamoxifen to tag part of the cells within the kidney with a fluorescent protein in a random manner. Podocyte renewal was then induced by the mean of transient TERT^ci^ overexpression. Examination of glomeruli at the end of the experiment allows to discriminate two potential cellular mechanisms supporting podocyte renewal: 1) activation of multiple podocyte progenitor cells, or 2) clonal expansion of a single progenitor cell. **b** Semi-thick (150–200 µm) kidney sections from UBC^CreERt2^;R26^Confetti^;actin-rtTA+ control mice collected 30 days (R30) after removing doxycycline. Scale bar = 100 µm. **c** Semi-thick kidney sections from UBC^CreERt2^;R26^Confetti^;i-TERT^ci^ mice collected 30 days (R30) after removing doxycycline, showing the invasion of single-colored clones (Cyan, left panel and Yellow, right panel), from the urinary pole (arrowheads) up to the glomerulus. Scale bars = 100 µm. **d** Semi-thick kidney section from an UBC^CreERt2^;R26^Confetti^;i-TERT^ci^ mouse collected 30 days (R30) after removing doxycycline, showing a clonal cell expansion that spreads from the proximal convoluted tubule (arrowhead) to the glomeruli. Scale bar = 50 µm. **e** Imaging of semi-thick kidney sections from UBC^CreERt2^;R26^Confetti^;i-TERT^ci^ mice collected 30 days (R30) after removing doxycycline, showing the emergence of single-colored glomeruli (from left to right, Yellow, Red, Green and Cyan). Scale bars = 50 µm. **f** Quantification of glomeruli and proximal tubules showing clonal expansions similar to panel (c), (d) and (e), in kidneys from UBC^CreERt2^;R26^Confetti^;actin-rtTA+ control (actin-rtTA + , *n* = 3), and UBC^CreERt2^;R26^Confetti^;i-TERT^ci^ mice (i-TERT^ci^, *n* = 3), 30 days after removing doxycycline. Data are shown for each animal and the mean value for each group is represented as a green line. For each animal, the total number of glomeruli (about 60) per kidney mosaic tiles were quantified. ***p = 0.0005 by t-test for i-TERT^ci^ versus control mice. **g** Whole-mount immunostaining for the nuclear marker of terminally differentiated podocyte WT1 (magenta) on semi-thick kidney sections from a UBC^CreERt2^;R26^Confetti^;i-TERT^ci^ mouse with nuclear GFP clonal glomeruli showing co-localization of the GFP + cells with WT1 (arrowheads).

To further dissect the mechanisms that conduct to podocyte renewal in the transient system of TERT^ci^ overexpression, we used an unbiased approach based on the ubiquitous and stochastic multi-color labeling of the mouse body cells. The R26^confetti^ mouse system enables the stochastic expression of membrane-targeted CFP (Cyan Fluorescent Protein), nuclear GFP (Green Fluorescent Protein), cytosolic YFP (Yellow Fluorescent Protein) or cytosolic RFP (Red Fluorescent Protein) upon Cre recombinase activation (Fig. 5a)^27^. This reporter mouse strain was crossed with the UBC^CreERt2^ mouse strain that drives ubiquitous recombination within the adult kidney upon tamoxifen treatment^28^. UBC^CreERt2^;R26^confetti^ mice were subsequently crossed with i-TERT^ci^ mice to perform an unbiased multi-lineage tracing study upon TERT^ci^-induced podocyte renewal (Fig. 5a). We reasoned that using sub-optimal recombination of this stochastic multicolor reporter would allow us to target a fraction of podocyte progenitor cells, and to subsequently trace them upon TERT^ci^-induced podocyte renewal (Fig. 5a). Using spectral confocal microscopy techniques, we assessed the recombination efficiency in the kidney of control mice and observed 30.9% (± 4.7%) of recombined cells in the medulla and 47% (± 8.6%) in the cortex (Supplementary Fig. 13). We then examined glomeruli once podocyte renewal was complete and normal kidney function resumed (R30) in i-TERT^ci^ mice. While the expression pattern of the four fluorescent proteins remained stochastic in glomeruli of UBC^CreERt2^;R26^Confetti^;actin-rtTA+ control mice (Fig. 5b), the appearance of clonal cell expansions were specifically observed in glomeruli of UBC^CreERt2^;R26^Confetti^;i-TERT^ci^ mice (Fig. 5c, d, e). Surprisingly, these glomerular clones appeared to emerge from the proximal convoluted tubular cells that shape the urinary pole of glomeruli (Fig. 5c, arrows). Interestingly, these clones were not observed beyond the proximal tubule, as these arrays of clonal cells were frequently interrupted in the proximal region by unlabeled or differently labeled cells (Fig. 5d, arrow). Such clonal expansions within the proximal convoluted tubules reaching the urinary pole of glomeruli were sparsely observed in UBC^CreERt2^;R26^Confetti^;actin-rtTA+ control mice, but rarely yield emergence of clonal glomeruli such as those observed in UBC^CreERt2^;R26^Confetti^;i-TERT^ci^ mice (Fig. 5f). Immunostaining for the nuclear podocyte differentiation marker WT1 on semi-thick kidney sections from a UBC^CreERt2^;R26^Confetti^;i-TERT^ci^ mouse with GFP + clonal glomeruli revealed that 29.4% (± 3.9%) of the GFP + cells displayed podocyte differentiation marker (Fig. 5g). This result suggests that a considerable proportion of the newly generated clonal cells are directed toward a podocyte fate. We next examined the color repartition of clonal glomeruli within each kidney section and observed an intriguing clustering of single-colored glomeruli in different territories of the cortex, suggesting the location of podocyte progenitors to be further than the proximal tubule (Supplementary Fig. 14). Altogether, our unbiased lineage-tracing methods concurred in supporting the existence of podocyte progenitor cells that get activated following TERT^ci^ expression pulse and that invade the proximal convoluted tubule up to the podocyte compartment in a clonal manner.

### Endogenous TERT is required for glomerular renewal following a TERT^ci^ pulse

**Fig. 6:**
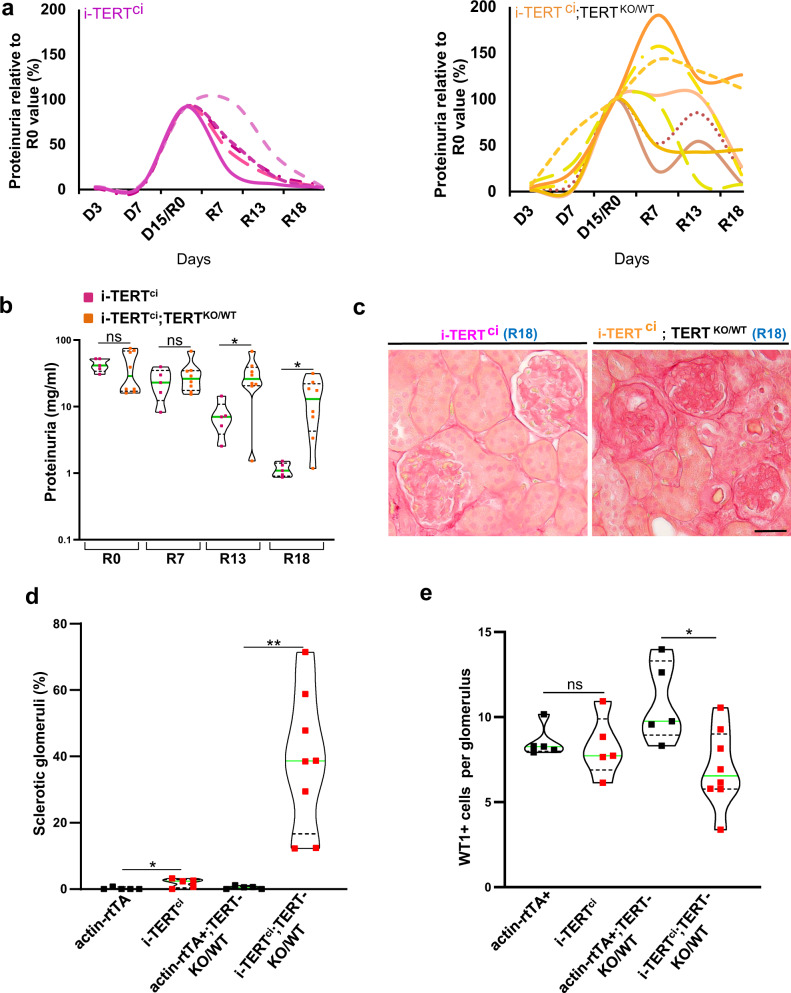
Endogenous TERT is required for glomerular renewal following a TERT^ci^ pulse. **a** Kinetic analysis by Bradford assay of proteinuria in urine samples of i-TERT^ci^ (pink, *n* = 5) and i-TERT^ci^;TERT^KO/WT^ (orange, *n* = 8) mice in the time course of transient TERT^ci^ overexpression experiment. Data are represented for each individual mouse as proteinuria level relative to R0 value in the time course of the experiment. **b** Proteinuria values (mg/ml) in i-TERT^ci^ (pink, *n* = 5) and i-TERT^ci^;TERT^KO/WT^ (orange, *n* = 8) mice during the reversal period. Data are shown for each animal and mean value for each group is shown as a green line. *p = 0.028 and *p = 0.023 by t-test for i-TERT^ci^;TERT^KO/WT^ versus i-TERT^ci^ at R13 and R18, respectively. **c** Sirius Red staining on kidney sections from i-TERT^ci^ and i-TERT^ci^;TERT^KO/WT^ mice collected 18 days after doxycycline withdrawal (R18). Scale bar = 50 µm. **d** Quantification of glomeruli with abnormal morphology (such as displayed for i-TERT^ci^;TERT^KO/WT^ mice in (c)), in kidney sections from actin-rtTA+ (*n* = 5), i-TERT^ci^ (*n* = 5), actin-rtTA+;TERT^KO/WT^ (*n* = 5) and i-TERT^ci^;TERT^KO/WT^ (*n* = 8) mice at 18 days of reversal (R18). Data are shown for each animal and the mean value for each group is shown as a green line. For each animal, all glomeruli (about 150) on the whole kidney section were analyzed. *p = 0.0306 and **p = 0.002 by *t-*test for i-TERT^ci^ versus actin-rtTA+ and for i-TERT^ci^;TERT^KO/WT^ versus actin-rtTA+;TERT^KO/WT^ respectively. **e** Quantification of the mean number of Wilms tumor protein (WT1) positive cells per glomerulus. Data are shown for each animal and the mean value for each group is shown as a green line. For each animal, all glomeruli (about 150) on the whole kidney section were analyzed. *p = 0.0136 by t-test for i-TERT^ci^;TERT^KO/WT^ versus actin-rtTA+;TERT^KO/WT^.

Our results show that endogenous TERT is required for glomerular repair following injury, and that a TERT^ci^ pulse is sufficient to trigger monoclonal expansion of progenitor cells in the adult kidney. Nonetheless, glomerular renewal observed in the i-TERT^ci^ mice only starts once TERT^ci^ overexpression is silenced. To determine whether endogenous TERT is involved in podocyte renewal observed in the i-TERT^ci^ mouse model, we generated i-TERT^ci^;TERT^KO/WT^ compound mice. We carried out transient TERT^ci^ overexpression in these mice and monitored proteinuria. The analysis of proteinuria in individual animals throughout the time course of the experiment strikingly revealed that 7 out of 8 i-TERT^ci^;TERT^KO/WT^ mice displayed perturbed proteinuria remission, and significantly higher proteinuria levels at reversal day 13 and at the end of the experiment at reversal day 18 when compared to i-TERT^ci^ mice (Fig. 6a, b). Further examination of kidney histology revealed massive sclerosis in glomeruli of i-TERT^ci^;TERT^KO/WT^ mice (Fig. 6c, d). We next carried out podocyte count in glomeruli by quantifying WT1 + nuclei per glomerular cross-section. Such analysis showed that while mean podocyte number per glomeruli remains preserved in i-TERT^ci^ after reversal, endogenous TERT invalidation in i-TERT^ci^;TERT^KO/WT^ mice results in 35% of podocyte loss (Fig. 6e). These results demonstrate the intriguing role of endogenous TERT in the remission of proteinuria and resolution of glomerular scarring in the i-TERT^ci^ model and prompted further molecular studies.

### TERT-induced podocyte renewal triggers modulation of genes involved in EMT, ECM remodeling, and KRAS signaling

**Fig. 7:**
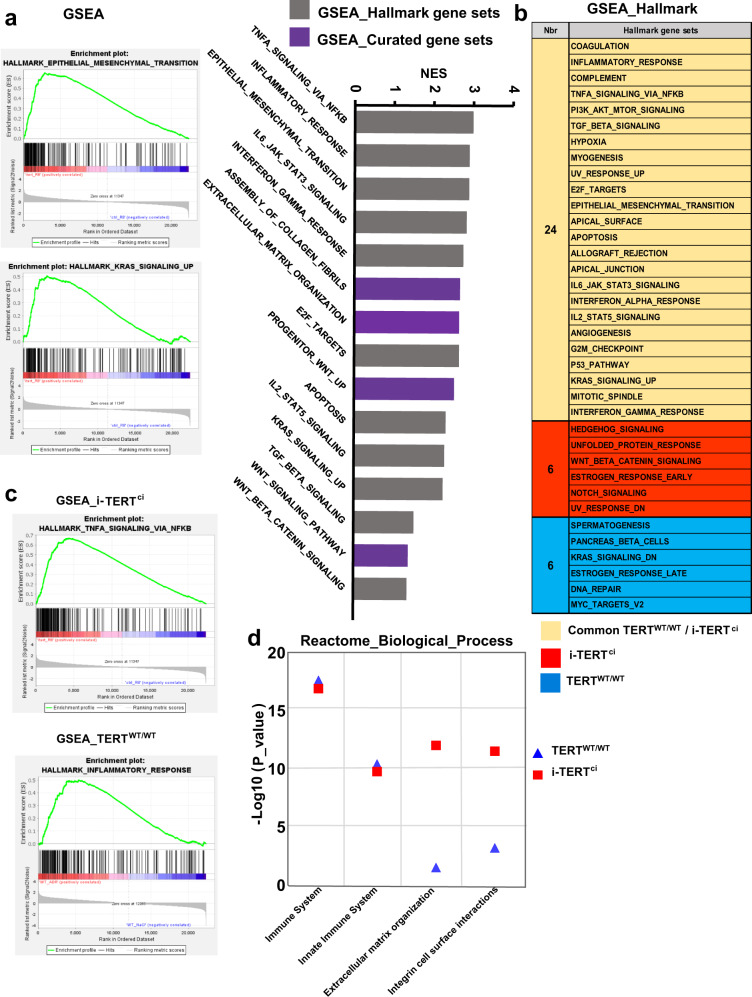
TERT-induced podocyte renewal triggers modulation of genes involved in EMT, ECM remodeling and KRAS signaling. **a** Top enriched gene signatures found by Gene Set Enrichment Analysis (GSEA) using Hallmark (grey histograms) and Curated (purple histograms) gene sets in kidneys of i-TERT^ci^ mice upon the recovery period (reversal day 8, R8). Enrichment profiles of Epithelial-to-Mesenchymal transition (EMT) and KRAS_UP signaling are shown on the left. **b** Comparison of the top 30 enriched gene signatures found by GSEA Hallmark upon podocyte renewal in TERT^WT/WT^ mice following ADR-induced injury (D18) and in i-TERT^ci^ mice (reversal day 8, R8). Common gene signatures between TERT^WT/WT^ and i-TERT^ci^ mice are highlighted in Yellow, gene signatures only enriched in i-TERT^ci^ mice are highlighted in red, and gene signatures only enriched in TERT^WT/WT^ mice are highlighted in blue. **c** GSEA enrichment profiles related to immune response in i-TERT^ci^ mice upon the recovery period (reversal day 8, R8) and in TERT^WT/WT^ following ADR-induced injury (D18). **d** Analysis of GO terms biological process using Reactome. The top 4 processes upregulated in i-TERT^ci^ mice upon the recovery period are shown (red squares), and further compared to the same processes in TERT^WT/WT^ mice (blue triangles).

Our results highlight the involvement of TERT non-canonical functions in glomerular renewal. To gain insight into the specific molecular targets and pathways activated in response to TERT^ci^ stimulation we performed high-throughput sequencing (bulk RNA-seq) analysis of total kidneys from i-TERT^ci^ mice. Kidneys were collected during the recovery period at reversal day 8 (R8), an early time point that corresponds to inflexion toward functional recovery of filtration function (Supplementary Fig. 15). Comparison of transcriptomes from i-TERT^ci^ vs. control actin-rtTA+ mice identified 1877 Differentially Expressed Genes (DEG) at R8 (Supplementary Fig. 15). GSEA analysis revealed enrichment of gene signatures including that of Epithelial-to-Mesenchymal Transition (EMT), extracellular matrix (ECM) remodeling, KRAS signaling, and several signatures related to inflammation (Fig. 7a). We next compared these gene signatures observed following a TERT^ci^ pulse to the gene signatures observed in a physiological regeneration context following ADR-induced injury (Fig. 7b). Such comparison highlighted six gene signatures specifically enriched in the TERT^ci^ regenerative context including Hedgehog, Wnt and Notch signaling (Fig. 7b, Supplementary Fig. 15). Interestingly, gene signatures related to inflammation were similarly enriched in both TERT^ci^ regeneration and ADR-induced physiological regeneration (Fig. 7b, c). Reactome pathway analysis confirmed similar strength of activation of the immune system in both regenerative contexts (Fig. 7d) and further revealed an increased induction of pathway related to ECM remodeling in TERT^ci^-enforced regeneration when compared to ADR-induced physiological regeneration (Fig. 7d). The high upregulation of ECM remodeling genes in i-TERT^ci^ mice was associated with specific upregulation of three metalloproteinases, namely MMP-23, MMP-9, and MMP-2 (Supplementary Fig. 15). Altogether, the molecular profiling of the signals triggered by TERT highlight the core pathways activated upon deployment of TERT pro-regenerative functions in the adult kidney (EMT, ECM remodeling, KRAS) and further suggest the involvement of specific pathways such as Hedgehog, Wnt and Notch in a context of enforced regeneration.

## Discussion

**Fig. 8:**
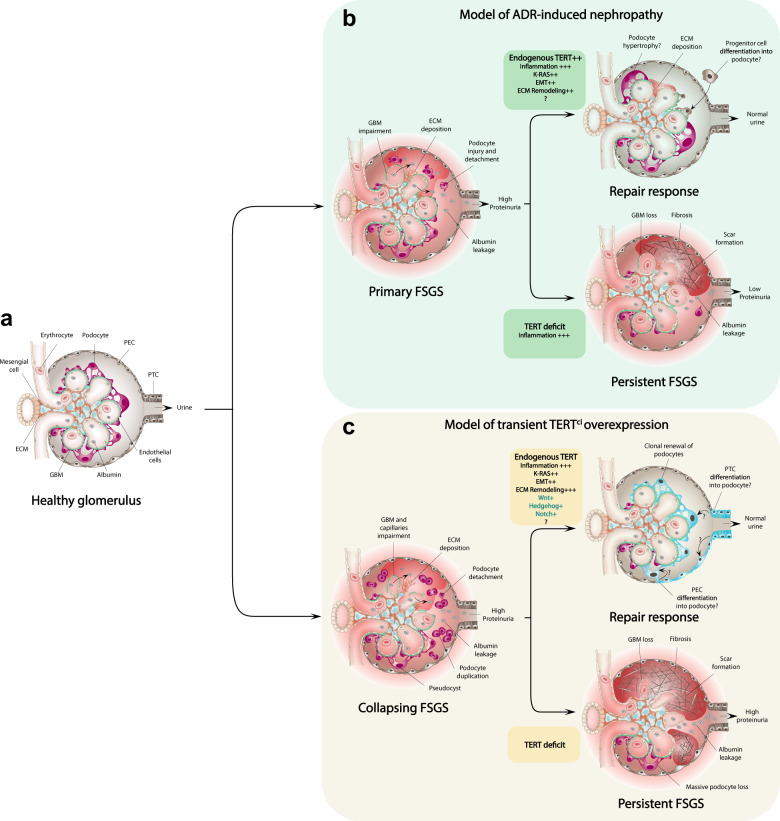
TERT exhibits non-canonical functions in glomerular repair and podocyte renewal in the adult mouse kidney. Model to illustrate the non-canonical functions of endogenous TERT and TERT^ci^ pulse in glomerular regeneration. **a** Healthy glomerulus showing terminally differentiated podocytes and intact glomerular basement membrane (GBM). **b** Following ADR-induced injury, podocytes insult conducts to their detachment from the glomerular capillaries and destabilization of the GBM. Albumin leakage in the urine leads to an increase of proteinuria. Two physiological responses can occur, a repair response that may involve putative podocyte hypertrophy and/or progenitor cell differentiation, or a scar formation that leads to persistent FSGS. Our results suggest that endogenous TERT could mediate the physiological repair via the activation of pro-EMT, ECM-remodeling and KRAS genes, while its deficit conducts to its failure. **c** In a model of ubiquitous and transient transgenic TERT^ci^ overexpression, the dramatic dedifferentiation and proliferation of podocytes conducts to a collapsing FSGS, characterized by the impairment of the GBM and capillary tuft collapse. Our results reveal two scenarios following TERT^ci^ reversal. The repair response is characterized by the expansion of podocyte progenitors that clonally invade the glomerulus to repopulate the PTC, PEC, and podocyte layers which is associated to the modulation of Wnt, Hedgehog, and Notch signaling. This functional repair that conducts to normalization of the proteinuria requires the bi-allelic expression of endogenous TERT. If endogenous TERT is deficient, the glomerular repair is not operating, leading to scar formation and persistent FSGS. Focal and segmental glomerulosclerosis (FSGS), Extracellular matrix (ECM), Parietal epithelial cells (PEC), Proximal tubule cell (PTC), Glomerular basement membrane (GBM), Epithelial-to-mesenchymal transition (EMT).

The main finding of this study is the uncovering of a key role of telomerase component TERT in glomerular repair following acute podocyte insult that conducts to normalization of proteinuria in the adult mammalian kidney. We first found that endogenous TERT is upregulated upon glomerular repair in a model of ADR-induced nephropathy, a process that involves the resolution of glomerular scarring and fibrosis that precludes regeneration (Fig. 8). The comparison of the deregulated genes identified in a bulk RNA sequencing of TERT^WT/WT^ and TERT^KO/KO^ total kidneys collected upon the physiological repair process further revealed that endogenous TERT regulates signals involved in Epithelial-to-Mesenchymal Transition (EMT), KRAS signaling, and remodeling of ECM components. Interestingly, the failure to repair glomeruli in the mouse model of *TERT* gene deficiency is observed in the first generation of TERT knock-out mice that do not show significant telomere DNA length shortening. More strikingly, heterozygous invalidation of the *TERT* gene also induced a dramatic failure of glomerular scarring remission, suggesting that non-canonical functions of telomerase are at play during kidney regeneration and that deployment of these functions rely on a fine dosage of TERT. We then used our previously published catalytic-dead telomerase (TERT^ci^) mouse model to explore the non-canonical roles of telomerase in podocyte renewal^12^. We found that a pulse of TERT^ci^ expression is sufficient to activate a progenitor cell population that clonally expend and repopulate the podocyte compartment. We demonstrated that such activation of progenitors required endogenous *TERT* expression, and was associated with the activation of signaling pathways similar to those identified in a physiological regeneration context. Additionally, TERT^ci^ overexpression triggered the expression of molecular signals reminiscent of the stem/progenitor gene signature, including Hedgehog, Wnt, and Notch signaling pathways. Our approaches thus identify TERT-dependent regenerative molecular actors in the adult mammalian kidney.

Homeostatic tissue repair following acute injury relies on a complex interplay between immune cells and ECM reorganization^29^. This phenomenon remains transient, and successful healing depends upon a step of efficient scar resolution that precedes cell replacement^30,31^. In certain chronic disease conditions, excess ECM deposition leads to fibrosis, a condition driving progressive loss of tissue function. In the adult kidney, podocyte effacement conducts to capillaries clogging by ECM proteins, an effective and immediate mechanism to limit massive leakage of the essential components of the bloodstream into the urine^32^ (Fig. 3b). However, unresolved accumulation of ECM components in glomeruli promotes glomerulosclerosis^33^, a condition that may be the consequence of inadequate regenerative response following successive injuries. Our results show that TERT plays critical functions in the regulation of genes involved in scar resolution upon kidney regeneration. Such functions of TERT on tissue ECM remodeling could be a pre-requisite for progenitor cells expansion to carry out tissue repair. The unbiased bulk RNA sequencing of total kidney cells approach uncovered the modulation of the three metalloproteinases MMP-24, MMP-12 and MMP-3. It is noteworthy that MMP-3 has been described as a target of hTERT non-canonical functions in vitro^34^. Further studies will be needed to decipher the molecular mechanism employed by TERT to modulate the expression of its target genes in a regenerative context.

While high levels of endogenous TERT have recently been shown to mark stem/progenitor cells in adult mice^35–37^, the pro-regenerative molecular signature of TERT in vivo remains elusive. Our data unveil the core pathways involved in TERT pro-regenerative functions in the adult kidney, and implicate a KRAS molecular signature. Interestingly, activating *KRAS* mutation was found to synergize with high levels of endogenous TERT to initiate pancreatic tumorigenesis^37^. Thus, we speculate that a fine interplay between TERT and KRAS signaling is critical to drive proper tissue renewal, and that balanced activation of those two is critical to avoid deleterious consequences such as cancer initiation. Additionally, we characterized the modulation of Hedgehog, Wnt, and Notch signaling pathways upon TERT^ci^-induced regeneration. These signaling pathways are established regulators of stem cell self-renewal, embryonic development, and differentiation, and their abnormal regulation can also conduct to tumorigenesis. While modulation of Wnt was previously shown to be involved in mediating TERT non-canonical functions^12,14,18^, the role of Hedgehog and Notch signaling in the deployment of TERT pro-regenerative functions remain to be explored.

A range of methods have been used in the adult mammals to identify potential podocyte progenitor cells. These strategies have led to the identification of several candidates for podocyte progenitor cells, which, depending on the study, are located within the glomerulus or within the juxtaglomerular apparatus^5–7,38,39^. The unbiased approach consisting in the stochastic multicolor tracing of random cells in the i-TERT^ci^ mice organism revealed that the adult kidney holds a population of cells capable of clonal expansion when submitted to the appropriate stimulus. The nature and localization of these cells remain enigmatic, however, we can speculate that the activation of these cells leads to the generation of podocyte progenitor cells that invade several glomeruli to restore the organ function. Interestingly, the co-localization of WT1 staining with the mono-colored glomerular cells indicates that the population of clonal cell progenitors can repopulate the podocyte layer but also potentially proximal tubular cells and other glomerular cell types such as the PEC. Several lines of evidences accumulated in this study suggest that the source of progenitor cell might represent adult stem cells, such as those described in other mammalian organs that reside within specialized microenvironments called niches^40^. However, it is noteworthy that recent studies conducted in organs with a well-defined stem cell niche, such as the skin and the intestine, demonstrated that the plasticity of differentiated cells significantly contribute to effective regeneration^41,42^. Thus, the podocyte progenitors might not emerge from a specific and dedicated cell type within its niche, but rather from cells that would display a striking and transient plasticity in response to transient TERT^ci^ expression. Further studies will be needed to unveil the origin of podocyte progenitor cells. Altogether, we consider that the i-TERT^ci^ mouse is a key model to further investigate the nature of podocyte progenitors.

Finally, a deficit of telomerase may prove to be involved in age-related renal dysfunctions, end-stage renal disease (ESRD) being an increasingly common organ failure in the aging population. Identification of pro-regenerative molecules targeted by telomerase in the adult kidney would offer new strategies that harness the organ’s intrinsic regenerative potential to delay or reverse renal disease progression.

## Methods

### Mice

Tetracycline-regulated i-TERT^ci^ transgenic mice, WT1^CreERt2^ mice (The Jackson Laboratory, stock# 010912), R26^mTmG^ mice (The Jackson Laboratory, stock# 007576), UBC^CreERt2^ mice (The Jackson Laboratory, stock# 008085), R26^confetti^ mice (The Jackson Laboratory, stock# 013731), and TERT^KO^ mice (The Jackson Laboratory, stock# 005423) were previously described^13,18,27,28,43–45^. Mice were PCR-genotyped using the following oligonucleotide pairs: 5’-CGCCCAGAAGCTTGGTGTAG−3’, 5’-GCTCCATGGCGATGACTTAG-3’ (actin-rtTA + ); 5’-GGATGTACTTTGTTAAGGCAGCA-3’, 5’-ACAACGGAGTTCCTCAGTGC-3’ (tetop-TERT^ci^ + ); 5’-ATCGCAGGAGCGGAGAAC-3’, 5’-GCAAACGGACAGAAGCATTT-3’ (WT1^CreERt2^); 5’-CTCTGCTGCCTCCTGGCTTCT-3’, 5’-CGAGGCGGATCACAAGCAATA-3’, 5’-TCAATGGGCGGGGGTCGTT-3’ (R26^mTmG^); 5’-GACGTCACCCGTTCTGTTG-3’, 5’-AGGCAAATTTTGGTGTACGG-3’ (UBC^CreERt2^); 5’-GAATTAATTCCGGTATAACTTCG-3’, 5’-AAAGTCGCTCTGAGTTGTTAT-3’, 5’-CCAGATGACTACCTATCCTC-3’ (R26^Confetti^); 5’-CCCCAGGCGCCGCACAAAGG-3’, 5’- GGTCCTGGCTGTTTTCTAAG-3’, 5’-CTGGATTCATCGACTGTGGC-3’ (TERT^KO^).

### Mice experiments

All mice were treated in accordance with the Institutional Animal Care and Use Committee approved guidelines at the Université Côte d’Azur (UCA, Nice, France) (CIEPAL-AZUR Agreements NCE/2012-32, NCE/2015-237#05225.03, APAFIS#2590-2015102215087555v3, APAFIS#16319-2018071917443610v2, and APAFIS#15232-2018051116515863-M20210803).

### Mouse model of ADR-induced podocyte injury

A single dose of 12 mg/kg of Adriamycin (ADR, Doxorubicin Hydrochloride, Sigma, Ref# D1515) or saline (NaCl 0.9%) was injected into the tail vein of 2-3 months old BALB/c mice or TERT knockout mice backcrossed for 10 generations with BALB/c mice (N10 BALB/c mice). For the TERT knockout experiment, TERT^WT/WT^ littermates were used as controls. For RNA-seq analysis, kidneys of TERT^KO/KO^ and TERT^WT/WT^ mice were collected 18 days after intravenous injection of ADR or saline (NaCl). All mice were weighed twice a week in the time course of the experiments, and urine was collected for each individual mouse twice a week. BALB/c mice were obtained from Janvier Lab (strain BALB/cJRj).

### Transient TERT^ci^ overexpression in i-TERT^ci^ mice

Double transgenic i-TERT^ci^ mice encompass both actin-rtTA+ and tetOp-TERT^ci^ + transgenes, allowing doxycycline-regulated and ubiquitous expression of TERT^ci^ in those mice. Those mice are maintained at a heterozygous status for each transgene. In order to induce TERT^ci^ overexpression in adult i-TERT^ci^ mice, doxycycline (2 mg/ml in 5% sucrose) (Doxycycline Hyclate, Sigma, Ref# D9891) was administered in drinking water in light-protected bottles and changed biweekly. The actin-rtTA+ control mice followed the same treatment than i-TERT^ci^ mice. High proteinuria levels (above 10 mg/mL) were observed in i-TERT^ci^ mice at about 15 days of doxycycline treatment (D15). At that time point, the doxycycline treatment was stopped (R0, mice switched to normal drinking water), and both i-TERT^ci^ and actin-rtTA+ control mice entered a reversal period during which proteinuria gradually regressed in i-TERT^ci^ mice. Only residual proteinuria persisted after 15 to 30 days of reversal (R15-30). For RNA-seq analysis, kidneys of i-TERT^ci^ and actin-rtTA+ control mice were collected 8 days after doxycycline withdrawal (reversal day 8, R8). All mice were weighed twice a week in the time course of the experiments, and urine was collected for each individual mouse twice a week.

### EdU pulse-chase experiment on i-TERT^ci^ mice

In this experiment, the actin-rtTA+ control mice followed the same treatments than i-TERT^ci^ mice. Doxycycline (2 mg/ml in 5% sucrose) was administered in drinking water in light-protected bottles and changed biweekly. High proteinuria levels (above 10 mg/mL) were observed in i-TERT^ci^ mice at about 15 days of doxycycline treatment (D15). At that time point, the doxycycline treatment was stopped (R0), and both i-TERT^ci^ and actin-rtTA+ control mice entered a reversal period during which proteinuria gradually regressed in i-TERT^ci^ mice. Once the doxycycline treatment was stopped (R0), EdU (0.4 mg/ml in 5% sucrose) (Life Technologies, Ref# E10415) was administered in drinking water in light-protected bottles and changed biweekly. The EdU treatment in drinking water was maintained for 8 days (R0-R8), and kidneys of the mice were either collected at the end of EdU treatment, 8 days after stopping doxycycline treatment (R8), or 15 days after stopping doxycycline treatment, which correspond to 7 days of EdU chase (R15). All mice were weighed twice a week in the time course of the experiments, and urine was collected for each individual mouse twice a week.

### Transient TERT^ci^ overexpression in WT1^CreERt2^;R26^mTmG^;i-TERT^ci^ mice and UBC^CreERt2^;R26^confetti^;i-TERT^ci^ mice

In these experiments, the WT1^CreERt2^;R26^mTmG^;actin-rtTA+ or UBC^CreERt2^;R26^confetti^;actin-rtTA+ control mice followed the same treatments than experimental mice. Intraperitoneal injection of (Z)−4-Hydroxytamoxifen (4OHT, Sigma, Ref# H7904; 1.5 mg in corn oil) was performed for 3 consecutive days. Doxycycline treatment was started 5 days after the last 4OHT injection. Doxycycline (2 mg/ml in 5% sucrose) was administered in drinking water in light-protected bottles and changed biweekly. High proteinuria levels (above 10 mg/mL) were observed in i-TERT^ci^ mice at about 15 days of doxycycline treatment (D15). At that time point, the doxycycline treatment was stopped (R0), and both i-TERT^ci^ and actin-rtTA+ control mice entered a reversal period during which proteinuria gradually regressed in i-TERT^ci^ mice. All mice were weighed twice a week in the time course of the experiments, and urine was collected for each individual mouse twice a week.

### Proteinuria analysis

Urine was collected from all individual mice before starting the experimental procedure, and twice a week in the time course of the experiment. Urine samples were assessed for protein content by the Bradford protein assay (BioRad, Ref# 5000002) and by the Albumin Creatinine Ratio Assay kit (Abcam, Ref# ab241018).

### Histology

Kidneys were fixed overnight in 10% buffered formalin and embedded in paraffin. Five μm tissue sections were stained with hematoxylin and eosin (H&E), or Masson Trichrome, or Sirius Red, or Periodic acid-Schiff for microscopic analysis.

### Immunohistochemistry

Antigen retrieval was performed on 5 μm paraffin sections using Vector unmasking reagent (Vector Laboratories, Ref# H3300). Mouse monoclonal primary antibodies were detected using a biotinylated anti-mouse IgG (MOM kit, Vector Laboratories, Ref# BMK-2202) followed by streptavidin-AlexaFluor647 (Jackson ImmunoResearch, Ref# 016–600). For immunostaining using rabbit, and chicken primary antibodies, kidney sections were blocked (PBS; 10 mg/ml BSA; 5% NGS; 0.01% Triton), then incubated with the primary antibody diluted in the blocking solution for overnight at 4 °C. For immunostaining using goat primary antibody, NGS was substituted by donkey serum in blocking solution. Detection was performed with a Cy3-conjugated goat anti-rabbit secondary antibody (Jackson Immunoresearch, Ref# 111–165), AlexaFluor488-conjugated goat anti-chicken secondary antibody (Jackson Immunoresearch, Ref# 103–545), and AlexaFluor594-conjugated donkey anti-goat secondary antibody (Thermo Fischer, Ref# A-11058). EdU was detected using Click-it Plus EdU AlexaFluor647 Imaging kit (Thermo Fisher, Ref# C10640).

The primary antibodies used were: Mouse Monoclonal Synaptopodin-specific (undiluted, Progen, Ref# 65194), Rabbit Monoclonal Ki67-specific (1:100, Spring Bioscience, Ref # M3062), Rabbit Monoclonal WT1-specific (1:100, AbCam, Ref# 89901), Chicken Polyclonal GFP-specific (1:300, Avès Labs, Ref# GFP-1020), Mouse Monoclonal AQP2-specific (1:100, Santa Cruz Biotechnology, Ref# sc-515770), Goat Polyclonal AQP2-specific (1:100, Santa Cruz Biotechnology, Ref# sc-9882), Mouse Monoclonal AQP1-specific (1:100, Santa Cruz Biotechnology, Ref# sc-25287), Mouse Monoclonal THP-specific (1:200, Santa Cruz Biotechnology, Ref# sc-271022), Rabbit polyclonal NCC-specific (1:100, Sigma, Ref# AB3553), biotinylated-LTL (1:200, Vector Laboratories, Ref# B1325).

### Quantitative RT-PCR

Snap-frozen tissues were ground with mortar and pestle. RNA was isolated from organs by homogenization in Trizol (Thermo Fischer, Ref# 15596018), and 1 µg of total RNA was reverse-transcribed (QuantiTect Reverse Transcription kit, Qiagen, Ref# 205311), then subjected to qPCR (FastStart Universal SYBR Green Master(Rox), Roche, Ref# 4913850001) using primer pairs specific for TERT, or HPRT (5’-GACTACTCAGGTTATGCCCAG-3’, 5’-TAGACCGTGACACTTCAACC-3’ for mouse TERT; 5’-TTGCTCGAGATGTCATGAAGGA-3’, 5’-CCAGCAGGTCAGCAAAGAACT-3’ for mouse HPRT). SYBR-green analysis was performed with the 7900HT Fast Real-Time PCR System machine (ABI). The expression level of TERT was normalized to the corresponding HPRT level.

### RNA in situ hybridization

Five μm thick paraffin kidney sections were processed for TERT mRNA in situ detection using the RNAscope 2.5 chromogenic Red detection kit (Advanced Cell Diagnostic), and counterstained with hematoxylin.

### Tissue preparation from Confetti mice

Semi-thick sections (200μm) from native kidneys were obtained using a vibrating microtome (Microm, Ref# HM650V). Freshly cut sections were fixed in 4% paraformaldehyde at room temperature for 10 min and washed in cold PBS. Sections were then mounted on slides in Mowiol, and imaged on a spectral confocal microscope (LSM880, Zeiss), calibrated beforehand.

### Whole-mount staining on semi-thick kidney sections

Semi-thick (200μm) fixed sections were blocked with TBS; 0.25% fish skin gelatin; 0.5% non-fat dry milk; 0.5% Triton, for overnight at room temperature under gentle shaking. The following day, the sections were incubated with primary antibody (rabbit monoclonal anti-WT1, AbCam, Ref# ab89901) diluted in blocking solution for overnight at room temperature under gentle shaking. The sections were then washed 3 times with PBS for 15 min, then five times with PBS; 0.5% Triton for 1 h each at room temperature under gentle shaking. Washed sections were incubated with the secondary antibody (AlexaFluor647-conjugated goat anti-rabbit, Jackson Immunoresearch, Ref# 111–605), diluted in the blocking solution for overnight at 4 °C under gentle shaking. Finally, the sections were washed 3 times with PBS for 15 min, then 5 times with PBS; 0.5% Triton for 1 h each at room temperature under gentle shaking, and mounted with Mowiol. Stained semi-thick kidney sections were imaged on a spectral confocal microscope (LSM880, Zeiss), calibrated beforehand. The percentage of glomerular GFP + cells that stain positive for WT1 was quantified on 10 independent glomeruli.

### Image analysis using ImageJ software

EGFP/Synaptopodin or EdU/nephron markers double-stained kidney sections were sequentially scanned for far-red and/or red and/or green signals allowing imaging of the entire section for each fluorophore. Sirius red-stained kidney sections were similarly scanned to acquire images of the entire section. For EGFP/Synaptopodin analysis, glomeruli areas were manually demarcated on entire kidney sections based on the synaptopodin positive structures. For Sirius red analysis, glomeruli areas were manually demarcated on entire sections based on histology. Demarcated glomeruli were defined as areas to be analyzed using ImageJ software. For EdU/nephron markers analysis, the total number of EdU positive nuclei on entire sections was determined by ImageJ. The signal inherent to nephron markers was then used to designate the areas to be subsequently analyzed. The number of EdU positive nuclei within these areas was then determined by ImageJ.

### RNA-seq

Total RNA was extracted from the whole kidney using the RNeasy Micro Kit with DNase treatment (Qiagen, Ref# 74004). RNA integrity was assessed using a Bioanalyzer 2100 (Agilent). Only samples with RNA Integrity Number (RIN) > 7 were further submitted to high throughput sequencing. Samples were subjected to high-output (2×100 bp) paired-end sequencing by BGI Global services (China). The quality of raw reads was assessed with FastQC. Clipping adaptor sequences were carried out using Trimmomatic and the trimmed reads were aligned to the mouse genome reference (mm10) downloaded from ENSEMBL using the STAR read aligner.

### Analysis of RNA-seq data

Differentially expressed genes (DEGs) were identified using the Bioconductor package DESeq2. DEGs were considered statistically significant at an adjusted p-value (*P*adj) < 0.05. DEGs were then sorted by log2 fold change (log2FC), and PCA and Heatmaps were plotted to check clustering and the homogeneity of the samples. Gene set enrichment analysis (GSEA) was performed using the hallmark C7 collections on GSEA software (version 3.0) and MsigDB database and C2 (curated gene sets) database. The reported GSEA output was selected based on a normalized enrichment score (NES) > 1.05. The Database for Annotation, Visualization, and Integrated Discovery (DAVID) was used to identify the enriched pathways using gene ontology (GO) terms. The g:Profiler tool set was used to identify the enriched pathways using the Reactome database.

### Statistical analysis

Data were analyzed and compared between groups using a two-tailed, unpaired Student’s *t*-test. A *p* < 0.05 was considered statistically significant and is presented as follow: * for *p* < 0.05, ** for *p* < 0.01, *** for *p* < 0.001.

### Reporting Summary

Further information on research design is available in the Nature Research Reporting Summary linked to this article.

## Supplementary information

Supplementary Files

REPORTING SUMMARY

## Data Availability

The RNA-seq data generated and analyzed during the current study have been deposited in NCBI’s Gene Expression Omnibus (GEO) repository (Edgar et al., 2002) and are accessible through GEO Series accession number GSE190978. The other datasets generated and analyzed during the current study are available from the corresponding author on reasonable request.
