# Supplementary material for: Telomerase is required for glomerular renewal in kidneys of adult mice
Source: NPJ Regen Med. 2022 Feb 11;7:15. doi: 10.1038/s41536-022-00212-z (PMC8837629; doi:10.1038/s41536-022-00212-z)
Supplement: Supplementary file 1 — Supplementary Files [file 41536_2022_212_MOESM1_ESM.pdf]

SUPPLEMENTARY FIGURES

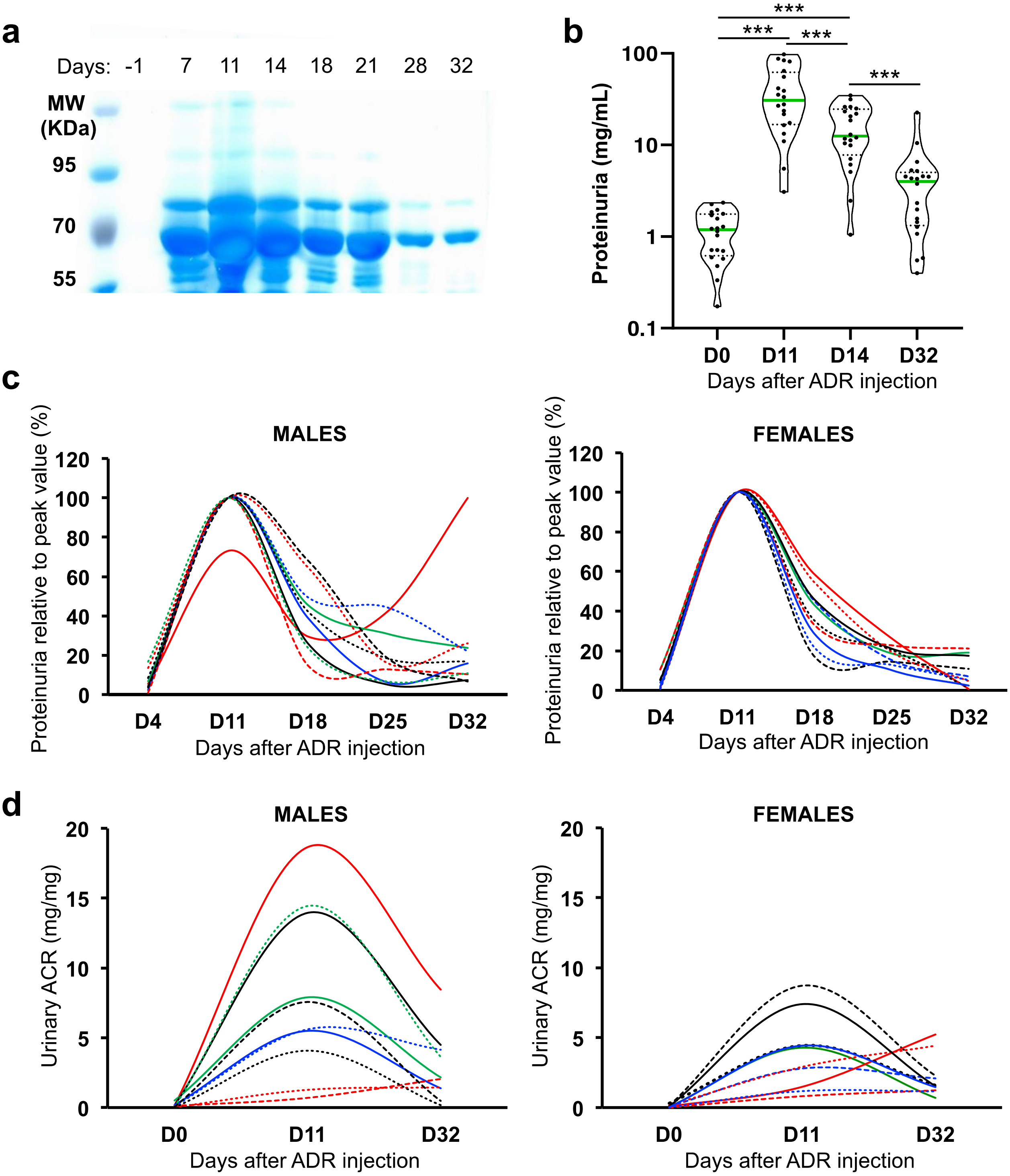

Supplementary Figure 1: Adriamycin-injected BALB/c mice display transient increase of proteinuria. **(a)** SDS-PAGE of urine samples collected from a BALB/c mouse 1 day before, and 7, 11, 14, 18, 21, 28 and 32 days after ADR injection. **(b)** Proteinuria monitoring of ADR-injected BALB/c mice (*n=20*) before injection (D0), 11 (D11), 14 (D14) and 32 (D32) days after injection. Data are shown for each animal in (mg/ml). Mean value for each time point is shown as a green line. p=4.79^{E-07} by t-test for D11 versus D0. p=3.25^{E-08} by t-test for D14 versus D0. p=0.0008 by t-test for D14 versus D11. p=1.74^{E-05} by t-test for D14 versus D32. **(c)** Kinetic analysis by Bradford assay of proteinuria in urine samples of 10 males and 10 female BALB/c mice. Data are represented for each individual mouse as proteinuria level relative to the peak value in the time course of the experiment. **(d)** Kinetic analysis of [albumin/creatinine ratio (ACR)] in urine samples of 10 males and 10 female BALB/c mice. Data are represented for each animal in (mg/mg) at day 0 (D0), day 11 (D11) and day 32 (D32).

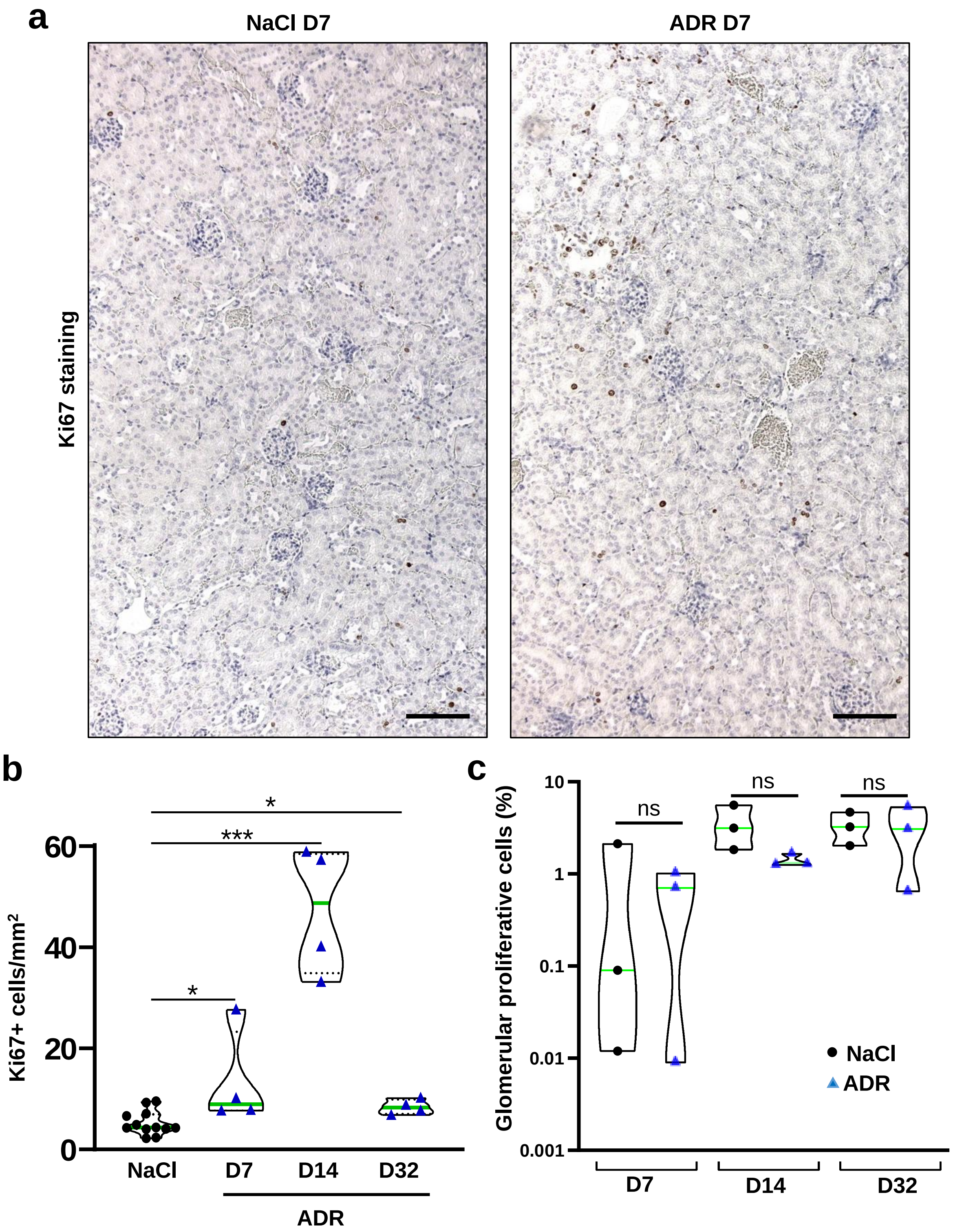

Supplementary Figure 2: Adriamycin-induced kidney injury triggers an increase in cell proliferation. **(a)** Immunostaining for the proliferation marker Ki67 in kidney sections from saline- (NaCl) and ADR-injected mice sacrificed 7 days after injection (D7). Scale bar=50µm. **(b)** Number of Ki67 positive cells per millimeter square in kidney sections from saline- (NaCl, n=12) and ADR-injected mice (ADR, n=4) collected 7 (D7), 14 (D14), and 32 (D32) days after injection. Data are shown for each animal. Mean value for each time point is shown as a green line. **p*=0.017 by t-test for NaCl versus ADR mice at D7. ****p*<0.001 by t-test for NaCl versus ADR mice at D14. **p*=0.028 by t-test for NaCl versus ADR mice at D32. **(c)** Percentage of nuclei that stain positive for Ki67 within glomeruli of entire kidney sections 7 (D7), 14 (D14) and 32 days (D32) after ADR injection.

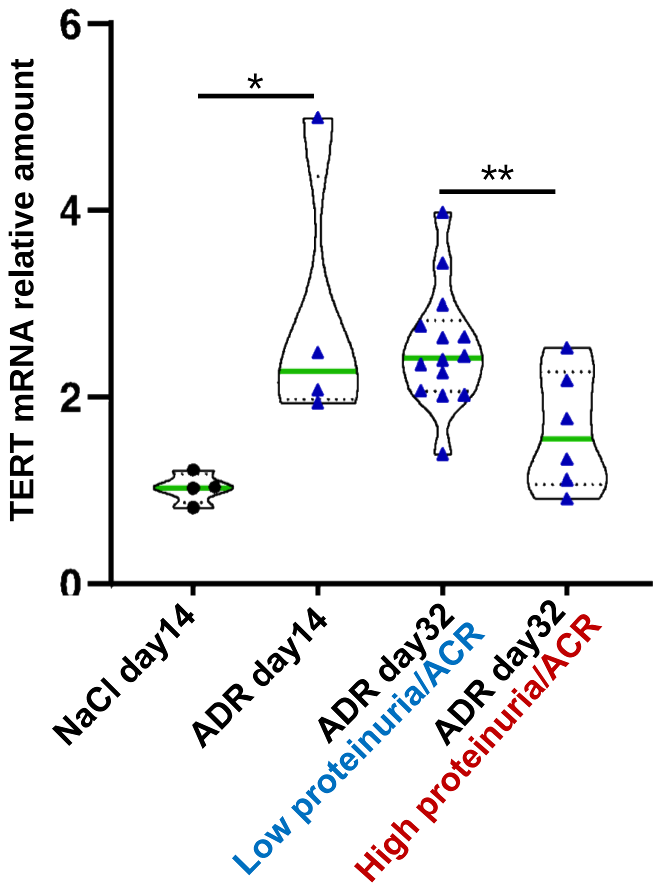

Supplementary Figure 3: Mice with persistent high proteinuria/ACR levels display lower levels of TERT expression than mice that recovered properly. TERT mRNA levels by qRT-PCR in whole kidneys from saline (NaCl, *n*=4) and ADR-injected mice collected 14 (*n*=4) or 32 days after injection. Mice at day 32 are clustered in two groups: (1) animals that display Low proteinuria/ACR at that time point (*n*=14), and (2) animals that display High proteinuria/ACR level at day 32 when compared to day 11 (*n*=6). Data are shown for each animal and mean value for each group is shown as a green line. The 6 animals with High proteinuria/ACR at day 32 show significantly lower levels of TERT mRNA when compared to the mice that display low proteinuria/ACR at day 32. *p=0.041 by t-test for ADR at day 14 versus NaCl at day 14. **p=0.005 by t-test for ADR at day 32 with High proteinuria/ACR versus ADR at day 32 with Low proteinuria/ACR.

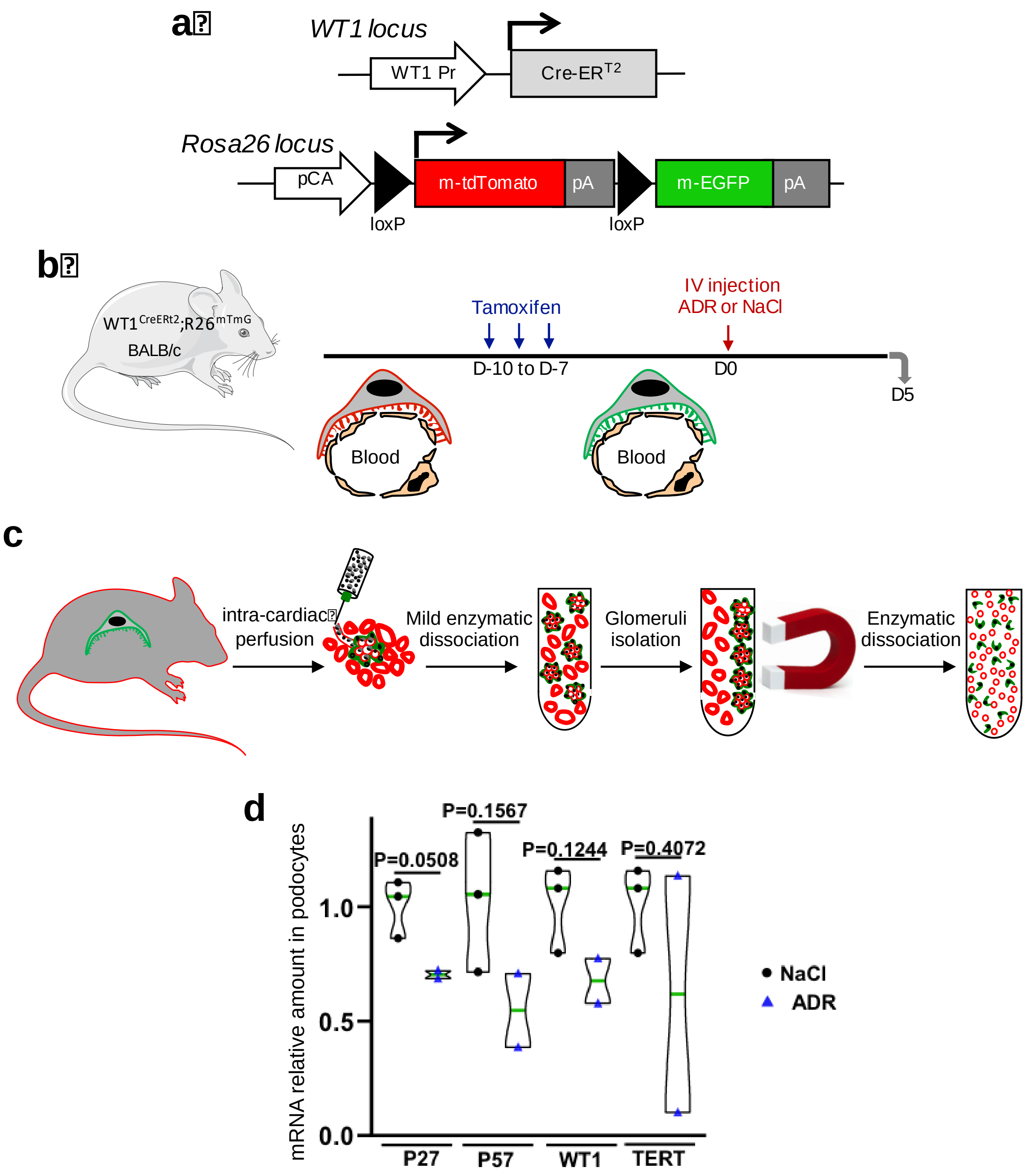

Supplementary Figure 4: Podocytes do not show increased TERT expression 5 days after ADR injection. (a-c) Experimental design to FACS sort podocytes from ADR- or saline-injected mice. **(a)** Schematic of the WT1^{CreERt2};R26^{mTmG} system. A CMV enhancer/chicken beta-actin core promoter (pCA) drives expression of a loxP-flanked membrane-targeted tdTomato cassette. Tamoxifen injection in WT1^{CreERt2};R26^{mTmG} mice leads to Cre-mediated DNA excision of the m-tdTomato coding sequence, and allows expression of the membrane-targeted EGFP in WT1 expressing cells, i.e. in mature podocytes in the adult kidney. **(b)** Schematic of the ADR-induced injury experiment in WT1^{CreERt2};R26^{mTmG} mice. WT1^{CreERt2};R26^{mTmG} BALB/c N10 mice were injected with tamoxifen during 3 consecutive days (D-10 to D-7) to permanently tag podocytes with EGFP, and the mice were injected 7 days later (D0) with ADR or saline (NaCl). Kidneys were then collected 5 days (D5) after ADR or saline injection. **(c)** Schematic of the method used to isolate podocytes. Glomeruli were first isolated by intra-cardiac perfusion of magnetic beads, followed by mild enzymatic dissociation of the tissue. Glomeruli filled with beads were then isolated using a magnet, and enzymatic dissociation allowed to obtain a single cell suspension of glomerular cells that was subjected to flow cytometry to sort podocytes. **(d)** Quantification of p27, p57, WT1 and TERT mRNA amounts in podocytes FACS-sorted 5 days after ADR or saline (NaCl) injection using the Biomark^{TM} HD System Analysis.

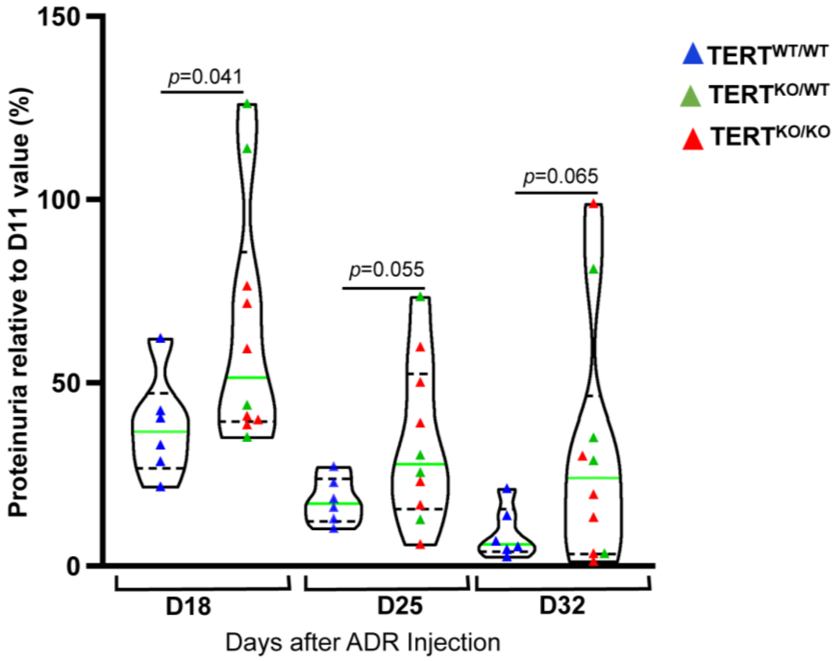

Supplementary Figure 5: TERT deficiency interferes with the kinetic of kidney filtration function recovery following ADR-induced injury. Analysis by Bradford assay of proteinuria after ADR injection of control mice ($TERT^{WT/WT}$, blue triangles), and TERT knockout heterozygote ($TERT^{KO/WT}$, green triangles), and homozygote ($TERT^{KO/KO}$, red triangles) mice. Proteinuria levels relative to day 11 (D11) values are represented for each individual animal. Mean value for each group is shown as a green line. *p* values obtained by t-test for TERT knockout versus control mice are shown.

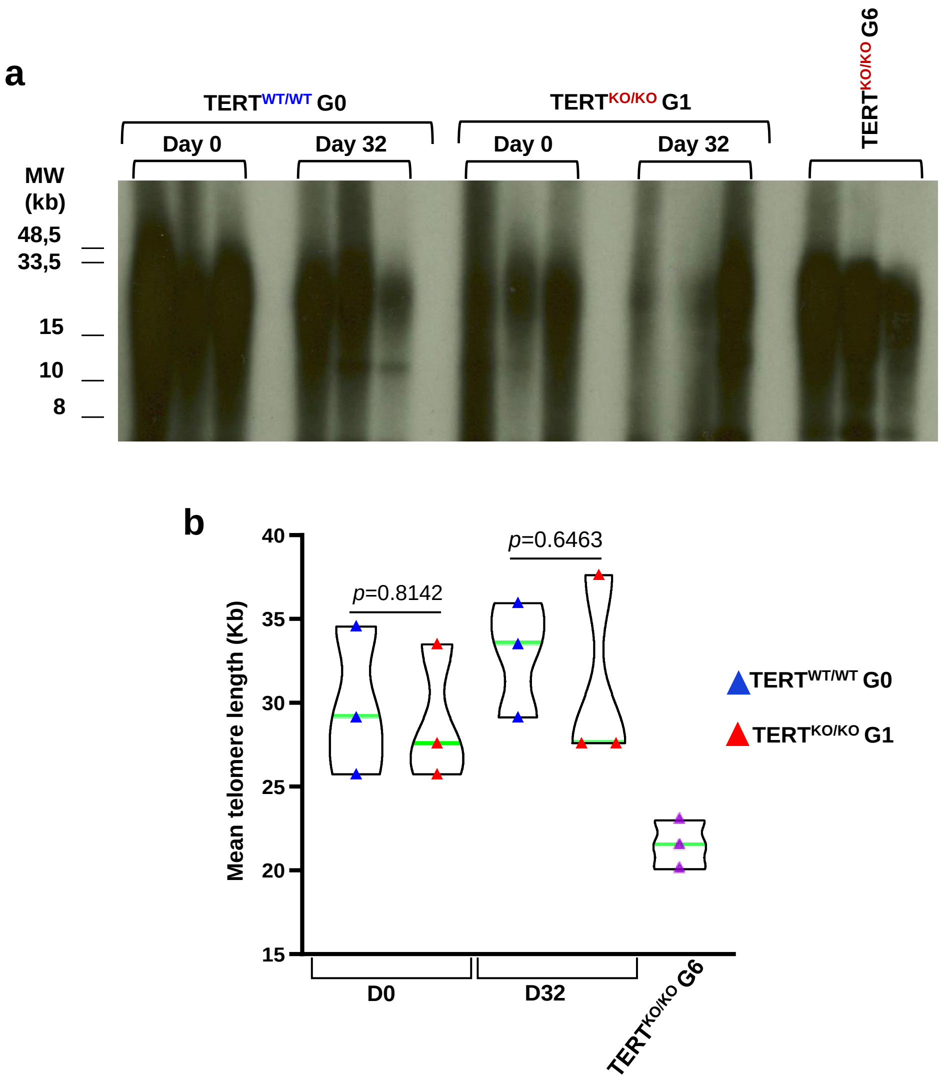

Supplementary Figure 6: Mean telomere length does not shorten following ADR-induced injury in kidney of TERT$^{WT/WT}$ and TERT$^{KO/KO}$ mice. (a) Telomere length analysis by Terminal Restriction Fragment (TRF) analysis on whole kidneys from three TERT$^{WT/WT}$ (generation 0, G0), and three TERT$^{KO/KO}$ (generation 1, G1) mice before ADR injection (day 0, D0) or 32 days after ADR injection (day 32, D32). TERT$^{KO/KO}$ mice that were interbred for 6 generations (G6) were used to assess the magnitude of telomere shortening in kidney of those mice that display the degenerative phenotypes characteristic of telomere dysfunction. **(b)** Quantification of data in **(a)**. Mean telomere length is represented for each individual animal. Mean value for each group is shown as a green line. *p* values obtained by t-test for TERT knockout versus control mice are shown.

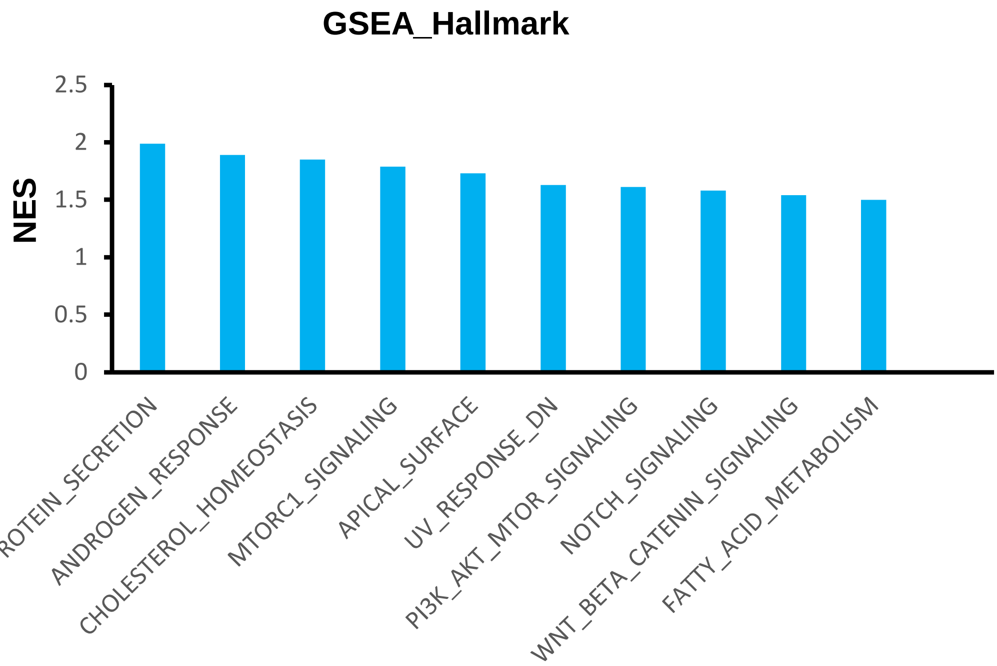

Supplementary Figure 7: Signaling pathways found enriched in kidneys of TERT$^{WT/WT}$ mice when compared to TERT$^{KO/KO}$ mice in saline conditions. Top 10 gene signatures identified by Gene Set Enrichment Analysis (GSEA) (Hallmark) in kidney of TERT$^{WT/WT}$ mice when compared to kidney of TERT$^{KO/KO}$ mice in control saline condition (NaCl). NES stands for normalized enrichment score.

DEG in $TERT^{WT/WT}$_ADR	log2FoldChange	padj	DEG in $TERT^{KO/KO}$_ADR	log2FoldChange	padj
MMP-27	6.3301	1.0654^{E-05}	MMP-27	6.8684	5.04^{E-05}
**MMP-24**	**3.2709**	**0.00046**	**MMP-24**	**-**	**-**
MMP-19	2.2253	1.3732^{E-05}	MMP-19	7.5036	0.0001
MMP-14	1.8554	1.531^{E-05}	MMP-14	2.0476	0.0105
MMP-13	-1.0075	0.00028	MMP-13	-1.6327	0.00018
**MMP-12**	**2.4077**	**4.1259^{E-06}**	**MMP-12**	**-**	**-**
**MMP-10**	**7.0228**	**7.2238^{E-08}**	**MMP-10**	**-**	**-**
MMP-8	3.8173	0.0025	MMP-8	4.0622	0.0318
MMP-7	6.1097	0.0001	MMP-7	3.4872	4.71^{E-05}
**MMP-3**	**3.3613**	**3.9151^{E-06}**	**MMP-3**	**-**	**-**

Supplementary Figure 8: TERT deletion prevents upregulation of several MMPs upon glomerular repair following ADR-induced injury. Tables showing all the MMPs found as deregulated genes (DEG) 18 days after ADR-injection in kidneys of $TERT^{WT/WT}$ mice (left) and of $TERT^{KO/KO}$ mice (right). Deletion of endogenous TERT prevents upregulation of MMP-24, MMP-12, MMP-10 and MMP-3 (bold) observed in normal regenerative conditions. For note, no MMPs are found deregulated in saline condition (NaCl) in kidneys of $TERT^{KO/KO}$ compared to $TERT^{WT/WT}$ mice.

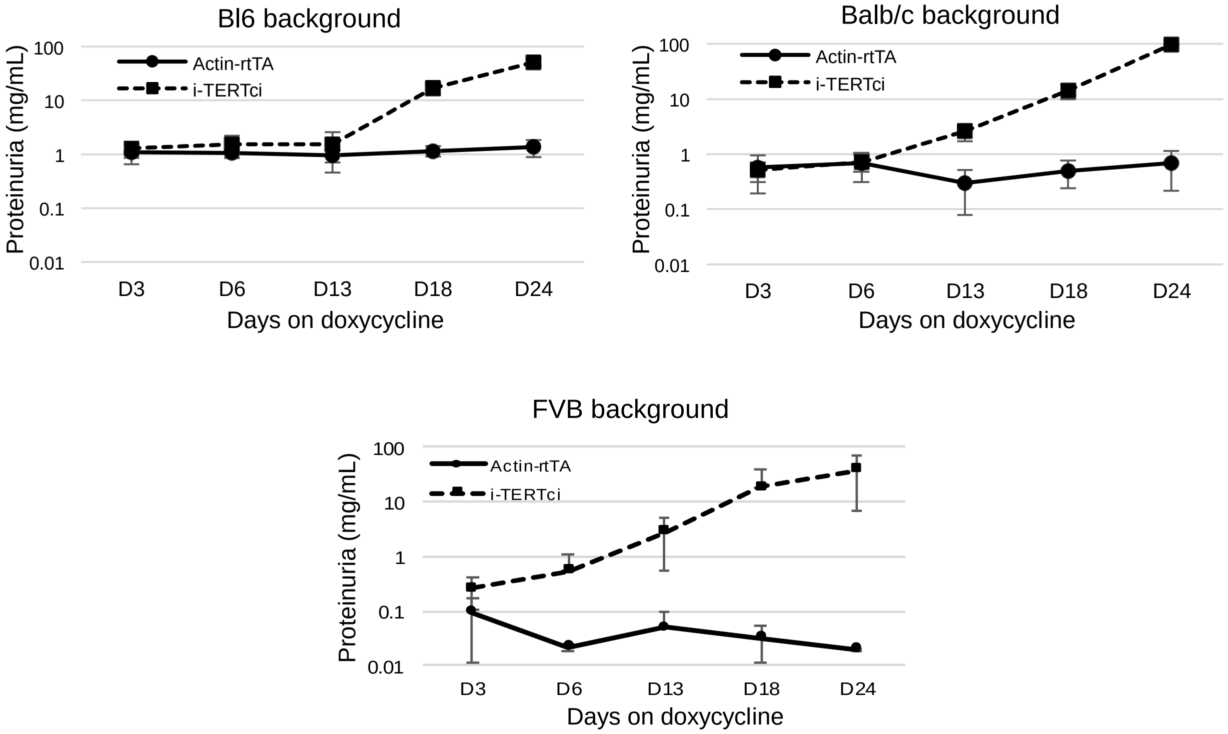

Supplementary Figure 9: Conditional overexpression of telomerase in adult mice induces kidney filtration dysfunction independently of mice genetic background. Kinetic analysis by Bradford assay of proteinuria in urine samples collected from single transgenic actin-rtTA+ (*n*=3) and i-TERT^{ci} (*n*=3) mice in Bl6 background (N1, top left), Balb/c background (N1, top right) and in FVB background (N3, bottom) treated with doxycycline for 24 days. Data are represented as mean ± SEM.

a

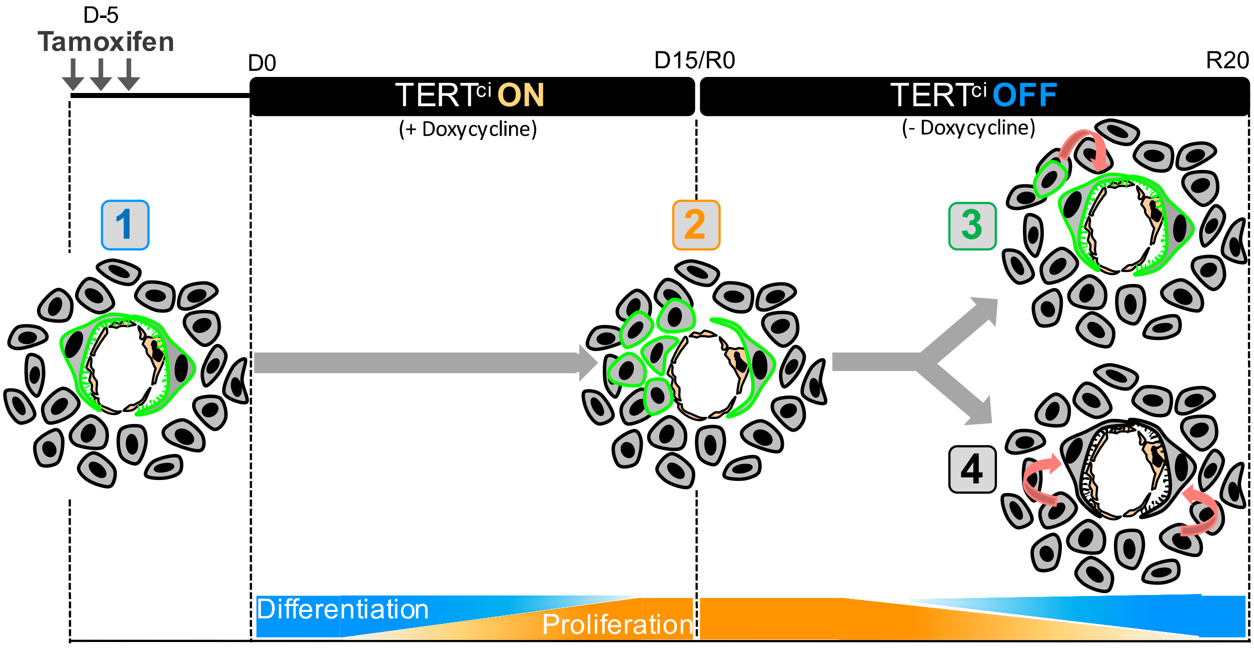

b

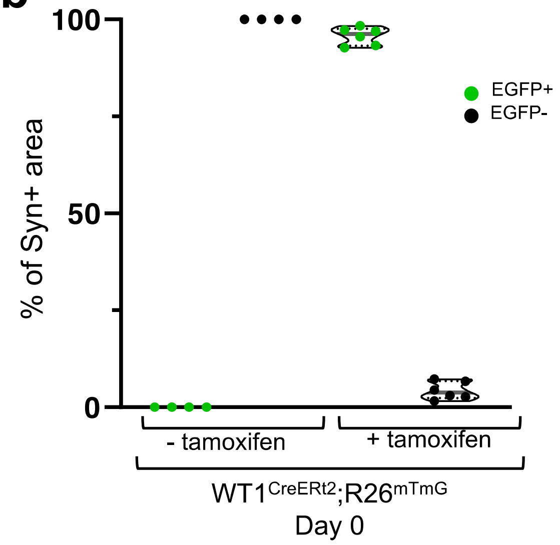

Supplementary Figure 10: Podocyte lineage tracing experiment upon TERT^{ci}-induced podocyte renewal. (a) Tamoxifen was injected in WT1^{CreERt2};R26^{mTmG};i-TERT^{ci} mice before starting the experiment to induce permanent EGFP tagging of mature podocytes (1). Podocytes were then activated by TERT^{ci} induction (TERT^{ci} ON) (2), and the mice were subsequently submitted to a reversal period (TERT^{ci} OFF) during which proteinuria gradually regressed. Prevalence of EGFP signal within differentiated podocytes was then assessed at the end of the reversal period. We reasoned that if we would observe no significant variation in the proportion of EGFP-tagged podocytes after reversal, then we could conclude that newly generated podocytes derived from initially tagged podocytes that have been transiently activated by TERT^{ci} (3). On the other hand, if we would observe differentiated podocytes that do not display EGFP after reversal, then we could conclude that these newly generated podocytes did not derive from initially tagged podocytes (4). **(b)** Percentage of Synaptopodin area that stain EGFP positive in mice treated (+tamoxifen) or not (-tamoxifen) with tamoxifen at day 0. For each animal, all the glomeruli (about 150) on the whole kidney section were analyzed. Data are shown for each animal. Results show that tamoxifen administration in WT1^{CreERt2};R26^{mTmG} control mice induces efficient EGFP tagging of mature podocytes with 95.7% of the Synaptopodin positive (Syn+) area that show EGFP signal.

a

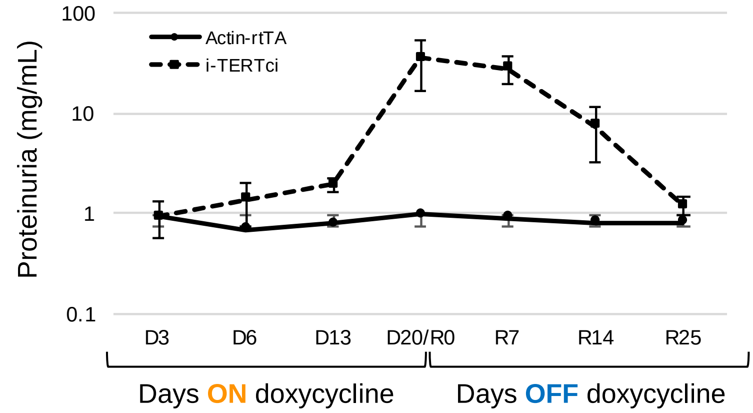

b

	actin-rtTA+ control mice	i-TERTci mice
Reversal day 8 (R8)	8178	10815
Reversal day 15 (R15)	4588	8880

Supplementary Figure 11: Dynamic of cell proliferation upon proteinuria remission in i-TERTci mice. (a) Kinetic analysis by Bradford assay of proteinuria from single transgenic actin-rtTA+ (*n*=3) and i-TERT^{ci} (*n*=3) mice treated with doxycycline for 20 days (D20), then reversed for 25 days (R25). Data are represented as mean ±SEM. **(b)** Average number of EdU+ cells on an entire kidney section for each group of mice (*n*=4 for actin-rtTA+ control mice and *n*=3 for i-TERT^{ci} mice).

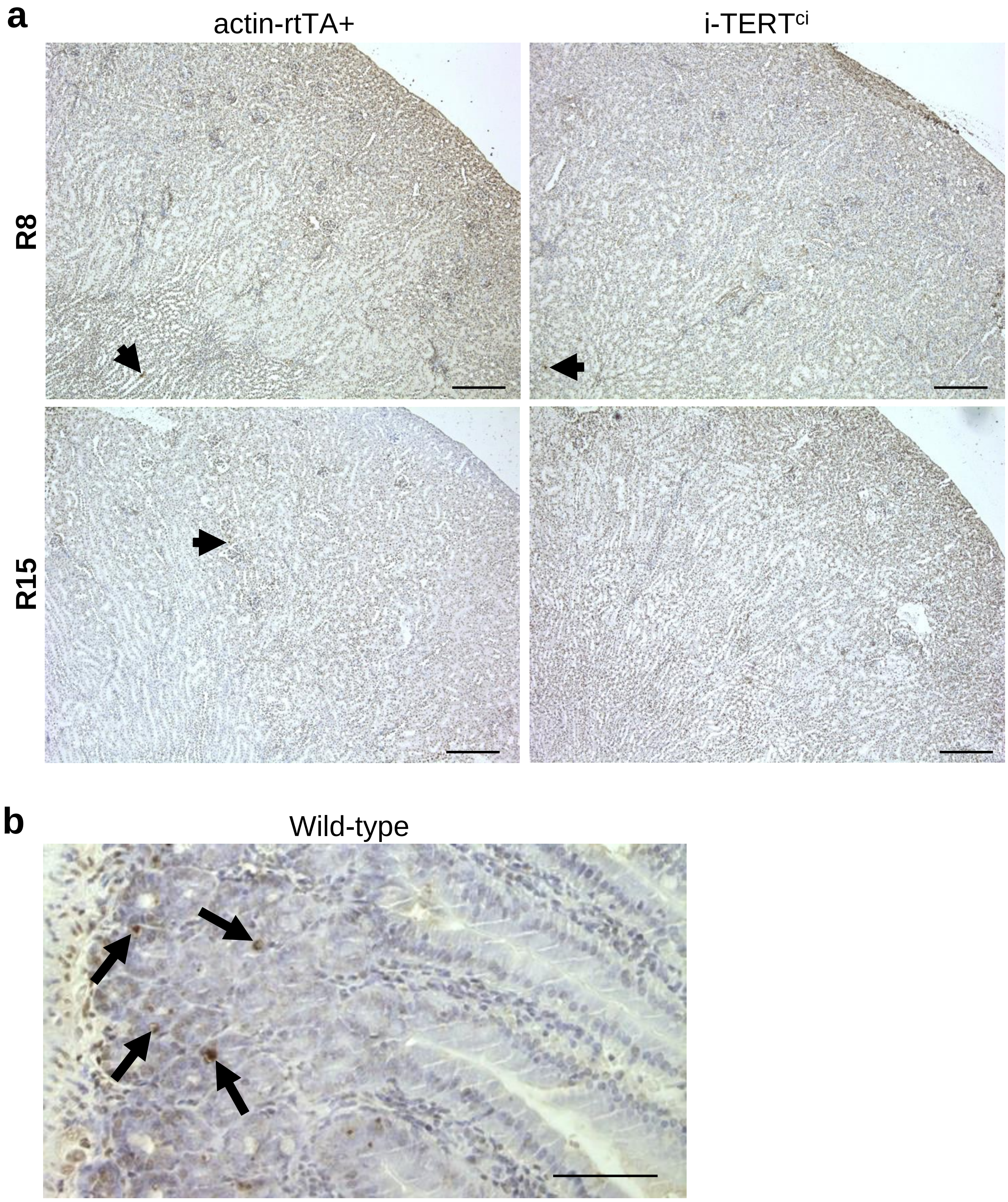

Supplementary Figure 12: TERT^{ci}-induced regeneration is not accompanied by apoptosis induction in kidney. (a) TUNEL assay in kidney sections from single transgenic actin-rtTA+ and i-TERT^{ci} mice treated with doxycycline for 20 days, and then reversed for 8 (R8) or 15 (R15) days. Scale bar = 300µm. No significant increase of TUNEL+ cells (black arrows) is observed in kidneys of i-TERT^{ci} mice at both time points. **(b)** Staining of intestine from a wild-type mouse was used as a positive control for the TUNEL assay. Scale bar = 100µm.

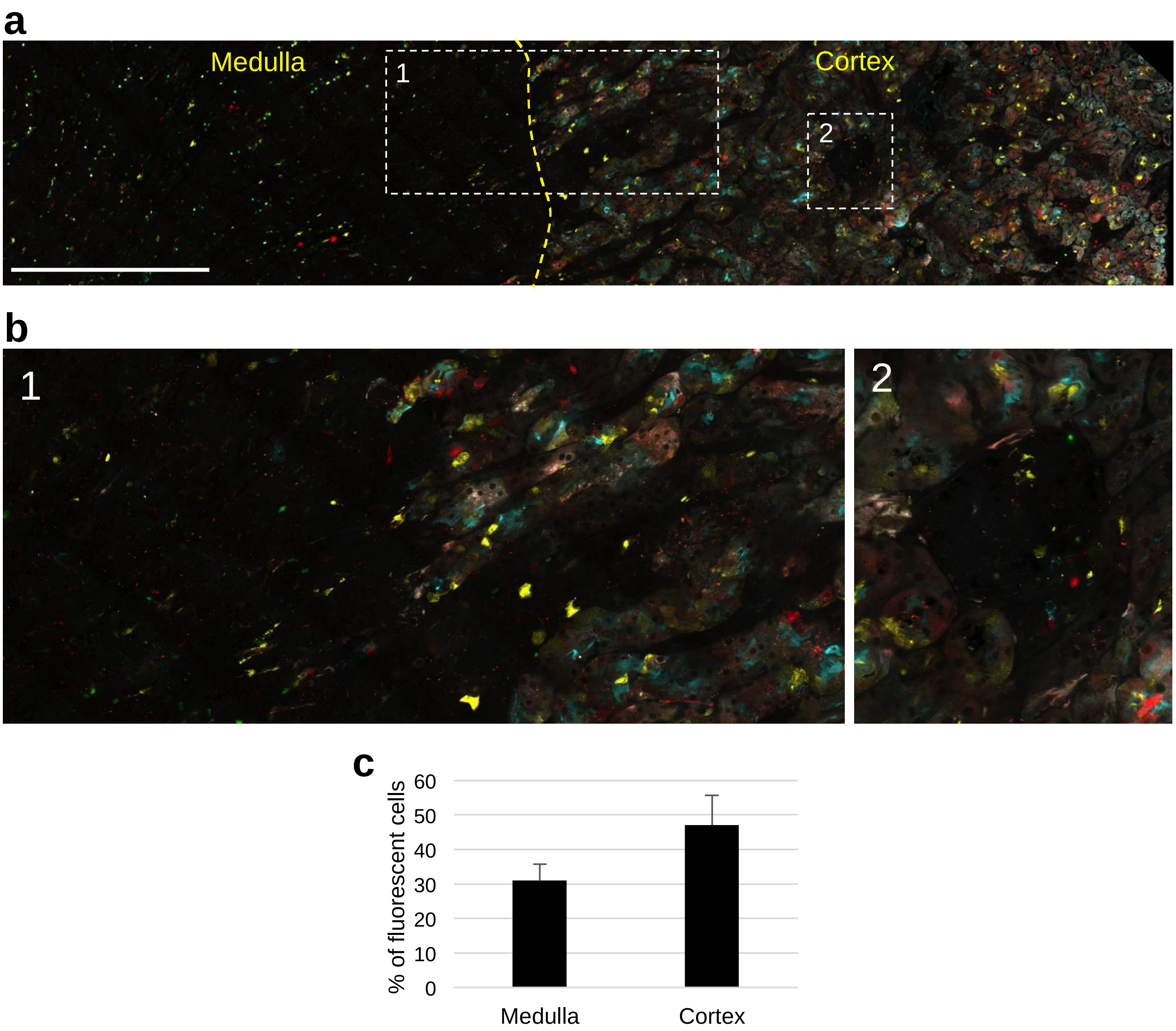

Supplementary Figure 13: Administration of suboptimal doses of tamoxifen in UBC^{CreERt2};R26^{Confetti} mice induces random and partial recombination of the Confetti cassette within the adult kidney. (a) Imaging of semi-thick kidney sections from UBC^{CreERt2};R26^{confetti} control mouse collected 5 days after the last tamoxifen injection. Scale bar = 500µm. **(b)** Magnifications of demarcated areas 1 and 2 highlighted in (a) showing lower efficiency of recombination in medullary tubules (1) and in glomeruli (2) when compared to cortical tubules. **(c)** Quantification of data in (a). Percentage of cells within the medulla and within the cortex that harbor expression of a fluorescent protein (*n=3*). Data are represented as mean ± SEM.

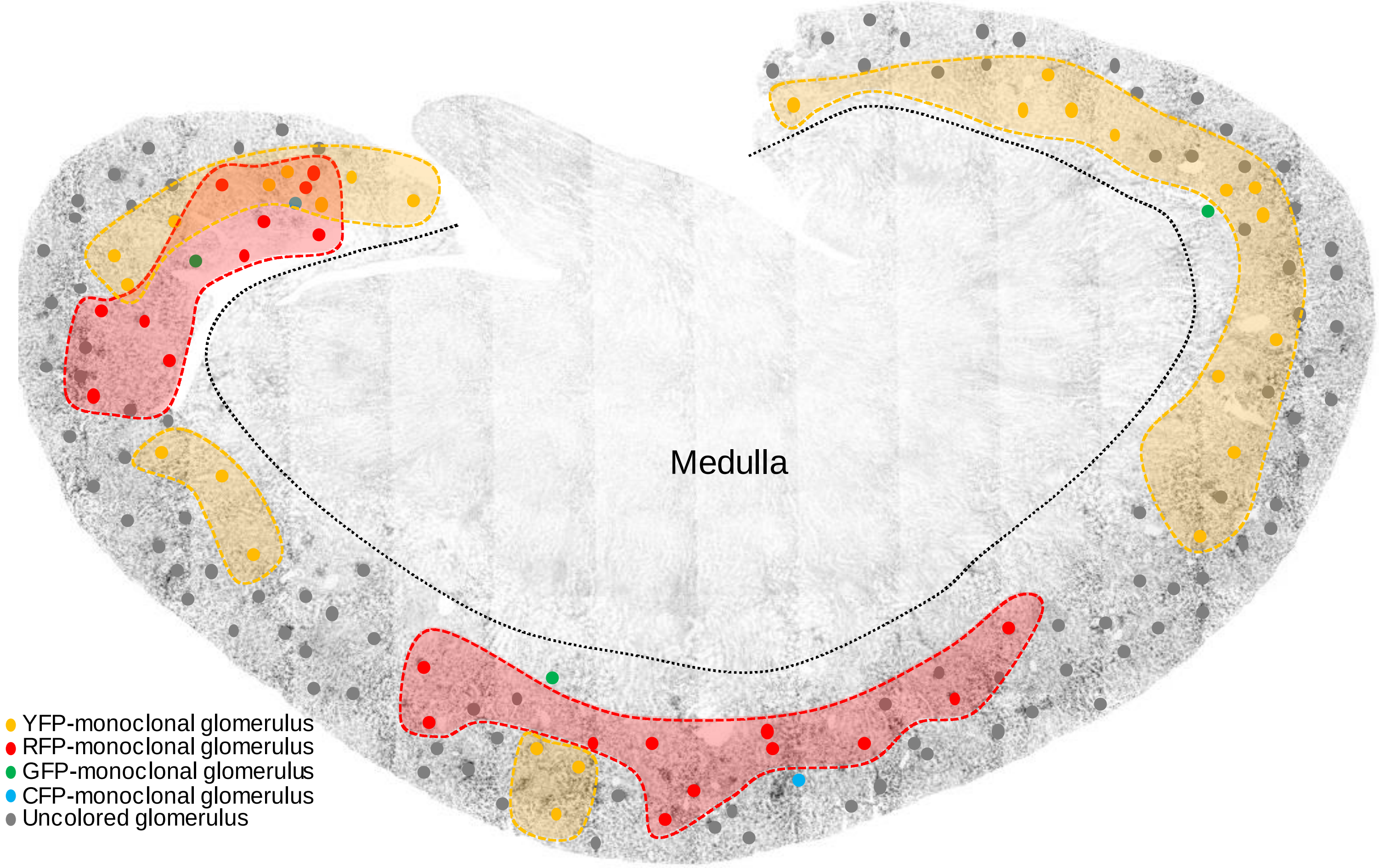

Supplementary Figure 14: Monoclonal glomeruli are color-clustered in territories within whole kidney sections. Representation of clonal glomeruli location within a semi-thick kidney section of a UBC^{CreERt2};R26^{confetti};i-TERT^{ci} mouse collected 30 days after removing doxycycline. While the color-balance varies between individuals, a similar pattern of color- clusters glomeruli was observed in the 2 other UBC^{CreERt2};R26^{confetti};i-TERT^{ci} mice examined.

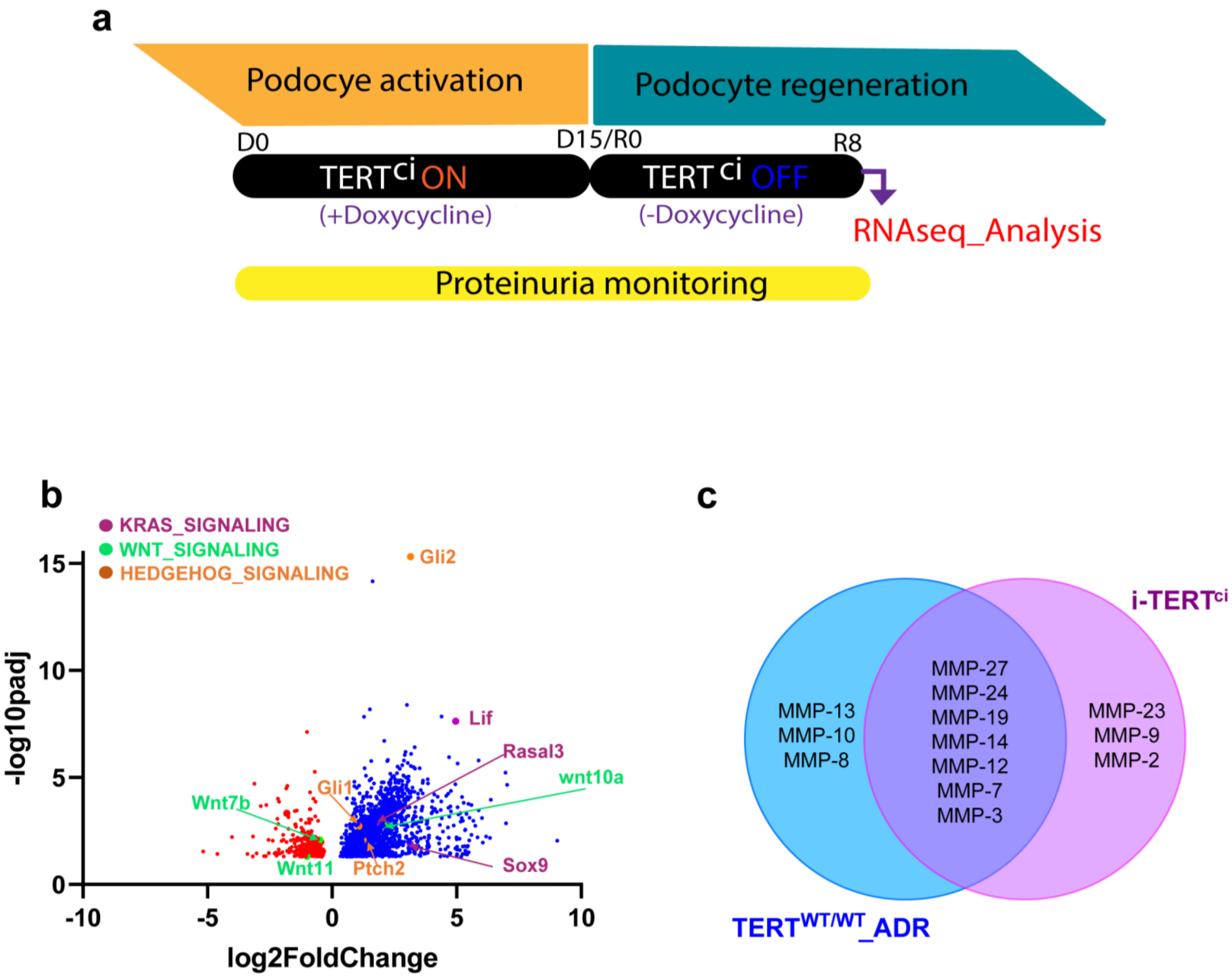

Supplementary Figure 15: Characterization of the molecular signature of TERT^{ci}-induced glomerular renewal. (a) Schematic representation of transient TERT^{ci} overexpression experiment performed for RNAseq analysis of twhole kidney. Kidneys were collected at reversal day 8, a time point associated to 40% (± 5%) of proteinuria improvement. **(b)** Volcano plot showing differentially expressed genes (DEG) in kidneys from i-TERT^{ci}_R8 mice compared to actin-rtTA+_R8 control mice. DEGs with adjusted p-value ≤ 0.05 are colored based on their expression. Blue and red dots indicate significantly up- and down-regulated genes respectively. Three representative genes associated to KRAS, WNT and HEDGEHOG signaling pathways are highlighted as magenta, green and orange dots respectively. **(c)** Venn diagram showing all MMPs found differentially expressed upon glomerular repair following ADR-induced injury in TERT$^{WT/WT}$ mice (blue) and following a TERT^{ci} pulse in i-TERT^{ci}_R8 mice (pink).

SUPPLEMENTARY METHODS

FACS sorting of podocytes from ADR-injected $WT1^{CreERt2}$;$R26^{mTmG}$ mice. Three months old, female $WT1^{CreERt2}$;$R26^{mTmG}$ BALB/c N10 mice were injected for 3 consecutive days with intraperitoneal injection of (Z)-4-Hydroxytamoxifen (4OHT, Sigma, Ref# H7904 ; 1.5 mg in corn oil). Seven days after the last tamoxifen injection, the mice received an intra-venous (IV) injection of ADR or saline as described above. Five days after IV injection, glomeruli were prepared as described by Takemoto et al., Am J Pathol, 2002. with slight adaptations. Briefly, intracardiac perfusion of heparin (100U/mL, Sigma, Ref# H6279), followed by perfusion of Dynabeads diluted in HBSS (Hanks' Balanced Salt Solution, Lifetech, Ref# H6648) was performed on anesthetized mice. Harvested kidneys were then minced into 1 mm3 pieces with a scalpel, and incubated in enzymatic digestion buffer (1 mg/mL collagenase IV, 0.2 mg/mL DNase I in HBSS) for 30 min at 37°C with gentle agitation. After digestion, all steps were carried out at 4°C or on ice. The digested kidneys were pressed through a 100µm cellstrainer. After spinning down the flow through, the pellet containing glomeruli was resuspended in 5mL HBSS, inserted into a magnetic particle concentrator, and the separated glomeruli were washed twice with HBSS. Glomeruli were then resuspended in 1 mL of enzymatic digestion buffer, and incubated for 30 min at 37°C with gentle agitation, and regular shearing was performed using a 25G needle. Single cells were then collected by centrifugation at 350g for 5 min at 4°C and resuspended in SEPBS (2% Fetal Bovine Serum, 2mM EDTA in PBS). The GFP+ cells were then sorted using a FACSAria III Cell Sorter (BD Bioscience), and the cells were harvested in RNA protect Cell Reagent (Qiagen, Ref# 76526). An average of 150,000 podocytes per mouse was obtained.

Detection of mRNA expression level using the Biomark™ HD System Analysis.
Reverse Transcription using the Fluidigm Reverse transcriptase. Extracted RNA were reverse transcribed using the Reverse Transcription Master Mix kit according to the manufacturer's instructions (catalog number # PN 100-6297). *cDNA pre-amplification step.* cDNAs were preamplified using the Preamp Master Mix kit (Fluidigm, cat. no. PN 100-5744) according to the manufacturer's instructions, combining 1ul of each 100 µM stock DELTAgene Assay, in a final volume of 200 µL. Thermal cycling conditions were: 95°C for 2 min followed by 15 cycles of 95°C for 15 s, 60°C for 2 min. After each preamplification reaction, samples were diluted 1:20 by adding 93 µL nuclease-free water. *Real time qPCR using Biomark™ HD System.* PCR was performed following Gene Expression using Delta Gene Assays protocol (Fluidigm PN 100-7717 B1). The pre-sample mix was prepared by mixing 2 µL SsoFast Evagreen Supermix with low ROX (2X) (Bio-Rad PN 1772-5211) and 0.2 µL Flexis Delta Gene Sample Reagent (Fluidigm PN 100-7673) and 1.8 µL preamplified cDNA to a final volume of 4 µL and then loaded into the Biomark™ IFC controller HX and transferred to the Biomark™ HD apparatus. Thermal cycling conditions were as follows: 50°C for 120 s, 95°C for 600 s followed by 40 cycles of 95°C for 15 s, 60°C for 1min.*Data analysis.* Real-time PCR data have been analyzed by the 2^-ddCT using HPRT as normalizer.

TUNEL assay. TUNEL analysis was performed on 5µm paraffin sections using TUNEL apoptosis detection kit (Millipore, Ref# S7100). Slides were deparaffinized, treated with proteinase K, quenched in 3% hydrogen peroxide and incubated with terminal deoxynucleotidyl transferase to label fragmented DNA with UTP-digoxigenin. Finally, sections were incubated with HRP-conjugated digoxigenin-specific antibody and visualized by chromogenic detection and hematoxylin counterstain.

Terminal Restriction Fragment (TRF) assay. Isolated DNA (6 ug for each sample) was digested overnight at 37°C using 80 units of *MboI* and *AluI* restriction enzymes (New England Biolabs). Those enzymes are frequent cutter and only digest non telomeric DNA, leaving telomeres intact. Digested DNA was then separated on a 1% agarose gel at 6V/cm for 13h with switch times ramped from 1 to 25 seconds (CHEF-DR® III Pulsed Field Electrophoresis System, BioRad 170-3690). The agarose gel was then washed with 0.25M HCl for 30 minutes for depuration, then twice with 0.5M NaOH (Sigma S8045) plus 1M NaCl (Promega H5273) for 20 minutes for denaturation, then twice with 1M Ammonium Acetate (Sigma A7262) for 20 minutes for neutralization. DNA was subsequently transferred from gel to a positively-charged nylon membrane (Amersham Hybond N+, GE HealthCare Life Sciences, RPN2222B) overnight in 20X SSC buffer (Euromedex, EU0300-C). The transferred DNA was fixed by UV crosslinking at 1200J. The cross-linked

membrane was then hybridized with the 32P-labelled telomeric probe overnight at 50°C. After hybridization the membrane was washed with buffer 1 (2X SSC) for 10 minutes at 50°C, then with buffer 2 (2X SSC, 1% SDS) for 30 minutes at 50°C, then twice with buffer 3 (0,2X SSC, 1% SDS) for 30 minutes each at 50°C. Telomere signals were detected following exposition to a phosphor screen for 6 hours, using a Phosphoimager system (GE Typhoon FLA 9500).
