# Supplementary material for: Telomerase is required for glomerular renewal in kidneys of adult mice
Source: NPJ Regen Med. 2022 Feb 11;7:15. doi: 10.1038/s41536-022-00212-z (PMC8837629; doi:10.1038/s41536-022-00212-z)
Supplement: Supplementary file 2 — REPORTING SUMMARY [file 41536_2022_212_MOESM2_ESM.pdf]

nature portfolio

Corresponding author(s): Marina SHKRELI

Last updated by author(s): Dec 17, 2021

Reporting Summary

Nature Portfolio wishes to improve the reproducibility of the work that we publish. This form provides structure for consistency and transparency in reporting. For further information on Nature Portfolio policies, see our Editorial Policies and the Editorial Policy Checklist.

Statistics

For all statistical analyses, confirm that the following items are present in the figure legend, table legend, main text, or Methods section.

n/a	Confirmed	
☐	☒	The exact sample size (n) for each experimental group/condition, given as a discrete number and unit of measurement
☐	☒	A statement on whether measurements were taken from distinct samples or whether the same sample was measured repeatedly
☐	☒	The statistical test(s) used AND whether they are one- or two-sided<br>*Only common tests should be described solely by name; describe more complex techniques in the Methods section.*
☐	☒	A description of all covariates tested
☐	☒	A description of any assumptions or corrections, such as tests of normality and adjustment for multiple comparisons
☐	☒	A full description of the statistical parameters including central tendency (e.g. means) or other basic estimates (e.g. regression coefficient) AND variation (e.g. standard deviation) or associated estimates of uncertainty (e.g. confidence intervals)
☐	☒	For null hypothesis testing, the test statistic (e.g. F, t, r) with confidence intervals, effect sizes, degrees of freedom and P value noted<br>*Give P values as exact values whenever suitable.*
☒	☐	For Bayesian analysis, information on the choice of priors and Markov chain Monte Carlo settings
☒	☐	For hierarchical and complex designs, identification of the appropriate level for tests and full reporting of outcomes
☒	☐	Estimates of effect sizes (e.g. Cohen's d, Pearson's r), indicating how they were calculated

Our web collection on statistics for biologists contains articles on many of the points above.

Software and code

Policy information about availability of computer code

Data collection	no software was used
Data analysis	Gene set enrichment analysis (GSEA), Database for Annotation, Visualization, and Integrated Discovery (DAVID), Reactome pathway analysis, FIJI

For manuscripts utilizing custom algorithms or software that are central to the research but not yet described in published literature, software must be made available to editors and reviewers. We strongly encourage code deposition in a community repository (e.g. GitHub). See the Nature Portfolio guidelines for submitting code & software for further information.

Data

Policy information about availability of data

All manuscripts must include a data availability statement. This statement should provide the following information, where applicable:

- Accession codes, unique identifiers, or web links for publicly available datasets
- A description of any restrictions on data availability
- For clinical datasets or third party data, please ensure that the statement adheres to our policy

The RNA-seq data generated in this study have been deposited in NCBI's Gene Expression Omnibus (GEO) repository and are accessible through GEO Series accession number GSE190978.
https://www.ncbi.nlm.nih.gov/geo/query/acc.cgi?acc=GSE190978
The access to the data is currently private, and we will make it public as soon as we get the green light from you.

Field-specific reporting

Please select the one below that is the best fit for your research. If you are not sure, read the appropriate sections before making your selection.

☒ Life sciences ☐ Behavioural & social sciences ☐ Ecological, evolutionary & environmental sciences

For a reference copy of the document with all sections, see nature.com/documents/nr-reporting-summary-flat.pdf

Life sciences study design

All studies must disclose on these points even when the disclosure is negative.

Sample size	Initial experiments were performed on 10 male + 10 female mice.<br>Due to high reproductibility of the results, subsequent experiments were performed on 3 to 4 mice per group, or maximum 10 when reviewers required it.
Data exclusions	no data were excluded from analysis
Replication	All the mouse experiments described in this manuscript have been performed multiple times with different experimenters within those last 5 years, and were extremely reproducible.
Randomization	Mice were allocated in groups based on their genotype. Control and experimental mice all followed the same treatments.
Blinding	All quantification and image analysis were performed blinded.

Reporting for specific materials, systems and methods

We require information from authors about some types of materials, experimental systems and methods used in many studies. Here, indicate whether each material, system or method listed is relevant to your study. If you are not sure if a list item applies to your research, read the appropriate section before selecting a response.

Materials & experimental systems

n/a	Involved in the study	
☐	☒	Antibodies
☒	☐	Eukaryotic cell lines
☒	☐	Palaeontology and archaeology
☐	☒	Animals and other organisms
☒	☐	Human research participants
☒	☐	Clinical data
☒	☐	Dual use research of concern

Methods

n/a	Involved in the study	
☒	☐	ChIP-seq
☒	☐	Flow cytometry
☒	☐	MRI-based neuroimaging

Antibodies

Antibodies used	Progen, Ref# 65194; Spring Bioscience, Ref# M3062; AbCam, Ref# ab89901; Aves Labs, Ref# GFP-1020; Santa Cruz Biotechnology, Ref# sc-515770; Santa Cruz Biotechnology, Ref# sc-9882; Santa Cruz Biotechnology, Ref# sc-25287 ; Santa Cruz Biotechnology, Ref# sc-271022 ; Sigma, Ref# AB3553 ; Vector Laboratories, Ref# B1325
Validation	All the antibodies used in this manuscript have been previously validated in the litterature

Animals and other organisms

Policy information about studies involving animals; ARRIVE guidelines recommended for reporting animal research

Laboratory animals	laboratory mice, both sexes, 2-4 months old
Wild animals	the study did not involve wild animals
Field-collected samples	the study did not involve samples collected from the field
Ethics oversight	All mice were treated in accordance with the Institutional Animal Care and Use Committee approved guidelines at the Université Côte d'Azur (UCA, Nice, France) (CIEPAL-AZUR Agreements NCE/2012-32, NCE/2015-237#05225.03, APAFIS#2590-2015102215087555v3, APAFIS#16319-2018071917443610v2, and APAFIS#15232-2018051116515863-M20210803).

Note that full information on the approval of the study protocol must also be provided in the manuscript.
